# A compendium of *Caenhorabditis elegans *regulatory transcription factors: a resource for mapping transcription regulatory networks

**DOI:** 10.1186/gb-2005-6-13-r110

**Published:** 2005-12-30

**Authors:** John S Reece-Hoyes, Bart Deplancke, Jane Shingles, Christian A Grove, Ian A Hope, Albertha JM Walhout

**Affiliations:** 1 Institute of Integrative and Comparative Biology, Faculty of Biological Sciences, School of Biology, University of Leeds, Woodhouse Lane, Leeds LS2 9JT, UK; 2 Program in Gene Function and Expression and Program in Molecular Medicine, University of Massachusetts Medical School, Worcester, 364 Plantation Street, Lazare Research Building, Room 605, MA 01605, USA

## Abstract

**Background:**

Transcription regulatory networks are composed of interactions between transcription factors and their target genes. Whereas unicellular networks have been studied extensively, metazoan transcription regulatory networks remain largely unexplored. *Caenorhabditis elegans *provides a powerful model to study such metazoan networks because its genome is completely sequenced and many functional genomic tools are available. While *C. elegans *gene predictions have undergone continuous refinement, this is not true for the annotation of functional transcription factors. The comprehensive identification of transcription factors is essential for the systematic mapping of transcription regulatory networks because it enables the creation of physical transcription factor resources that can be used in assays to map interactions between transcription factors and their target genes.

**Results:**

By computational searches and extensive manual curation, we have identified a compendium of 934 transcription factor genes (referred to as wTF2.0). We find that manual curation drastically reduces the number of both false positive and false negative transcription factor predictions. We discuss how transcription factor splice variants and dimer formation may affect the total number of functional transcription factors. In contrast to mouse transcription factor genes, we find that *C. elegans *transcription factor genes do not undergo significantly more splicing than other genes. This difference may contribute to differences in organism complexity. We identify candidate redundant worm transcription factor genes and orthologous worm and human transcription factor pairs. Finally, we discuss how wTF2.0 can be used together with physical transcription factor clone resources to facilitate the systematic mapping of *C. elegans *transcription regulatory networks.

**Conclusion:**

wTF2.0 provides a starting point to decipher the transcription regulatory networks that control metazoan development and function.

## Abstract 2

A compendium of 934 transcription factor genes in *C. elegans* and identified by computational searches and extensive manual.

## Background

Metazoan genomes contain thousands of predicted protein-coding genes. During development, pathology, and in response to environmental changes, each of these genes is expressed in different cells, at different times and at different levels. Spatial and temporal gene expression is controlled transcriptionally through the action of regulatory transcription factors (TFs) [1,2]. Transcription of each gene can be up- or down-regulated by TFs that bind to *cis*-regulatory DNA elements. These elements include upstream elements located in the proximal promoter, and enhancers or silencers that can be located at a greater distance from the transcription start site. Frequently, the expression level of a gene is the result of a balance between transcription activation and repression governed by multiple *cis*-regulatory elements and, hence, multiple TFs. The combinatorial nature of gene transcription provides an exquisite level of flexibility to regulate genome expression.

**Figure 1:**
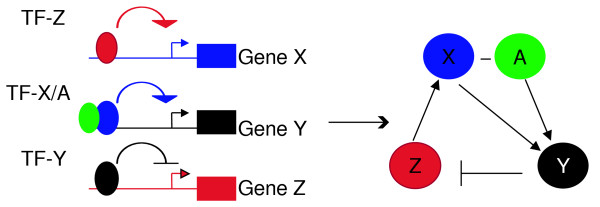
Transcription regulatory networks provide models to understand differential gene expression at a systems level. Transcription regulatory networks are composed of two types of components, or nodes: the genes involved in the system and the TFs that regulate their expression. Protein-protein interactions between TFs and protein-DNA interactions between TFs and their target genes can be visualized in transcription regulatory networks. The dashed line represents TF-TF protein-protein interaction (heterodimer). Arrows represent protein DNA interactions that result in transcription activation; the blunt 'arrow' represents protein-DNA interaction that results in repression of transcription.

The understanding of differential gene expression at a genome-wide, or systems, level has been greatly facilitated by the mapping and analysis of transcription regulatory networks (Figure 1) [3-5]. Such networks are composed of two types of components, or nodes: the gene targets that are subject to transcriptional control and the TF proteins that execute transcriptional control. Whereas transcription regulatory networks have been extensively studied in relatively simple unicellular systems, they remain largely unexplored in complex, metazoan systems.

The nematode *Caenorhabditis elegans *is a powerful model to decipher metazoan transcription regulatory networks. The *C. elegans *genome has been completely sequenced and is predicted to contain 19,735 protein-coding genes (WormBase WS140)[6]. Several functional genomic resources enable the systematic dissection of differential gene expression at a systems level and in a high-throughput manner. For instance, microarrays are available to investigate temporal and, to a certain extent, spatial gene expression levels [7-9]. In addition, *C. elegans *'ORFeome' [10] and 'Promoterome' [11] resources provide open reading frame (ORF) and promoter clones, respectively. These clones can be used for a wide variety of experiments that aim to dissect transcription regulatory networks (see below).

With seven years of progressive refinement of the genome annotation since the publication of the genome sequence [12-14], comprehensive predictions of protein-coding genes are available. However, there is no up-to-date compendium of predicted *C. elegans *TFs. Several lists of putative *C. elegans *TFs have been generated previously (Table 1; we refer to this combined set as wTF1.0 (worm transcription factors version 1.0)), but none of these are comprehensive or readily accessible. The earliest lists were created by scanning sequences of *C. elegans *proteins, as predicted from the genome annotation, for well-defined DNA binding domains: Hobert and Ruvkun [15] focused on homeodomain, paired domain, T-box, basic helix-loop-helix (bHLH), basic region leucine zipper (bZIP), Fork Head, erythroblast transformation specific (ETS) and nuclear hormone receptor (NHR) proteins; and Clarke and Berg [16] focused on various zinc-finger proteins. For comparative genomic studies primarily focusing on *Drosophila melanogaster *[17] or *Arabidopsis thaliana *[18], a list of predicted *C. elegans *proteins containing various DNA binding domains was compiled. While several (sub)-families of *C. elegans *TFs have been studied in greater detail (for example, bHLH [19], CUT homeodomain [20], and DM zinc-finger [21]), the most recent list of "all *C. elegans *TFs" was compiled five years ago when Riechmann and colleagues [18] scanned WormPep 20 (19,101 proteins). During the past few years, the creation of improved computational tools [13] and the completion of the *C. briggsae *genome sequence [14] have enabled a great improvement in the annotation of the *C. elegans *genome. Here, we used a combination of bioinformatics and extensive manual curation to generate wTF2.0, a comprehensive compendium of predicted *C. elegans *TF genes. We discuss how wTF2.0 can be used together with physical ORFeome and Promoterome clone resources to decipher transcription regulatory networks that control metazoan differential gene expression at a systems level.

## Results and discussion

### TF predictions: Gene Ontology term-based searches

**Figure 2:**
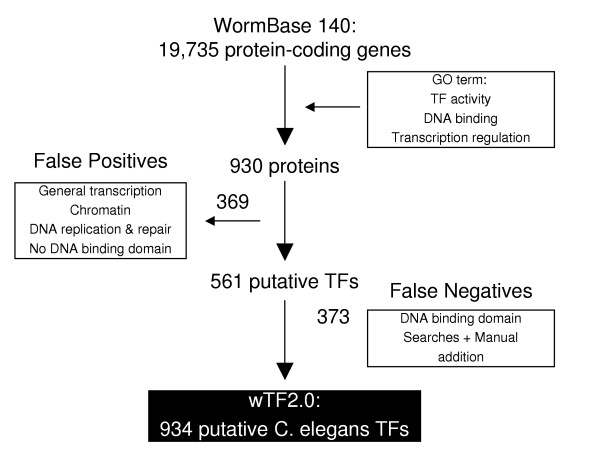
Generation of wTF2.0, a comprehensive compendium of *C. elegans *TFs. Schematic overview of the wTF2.0 generation pipeline. See main text for details.

**Figure 3:**
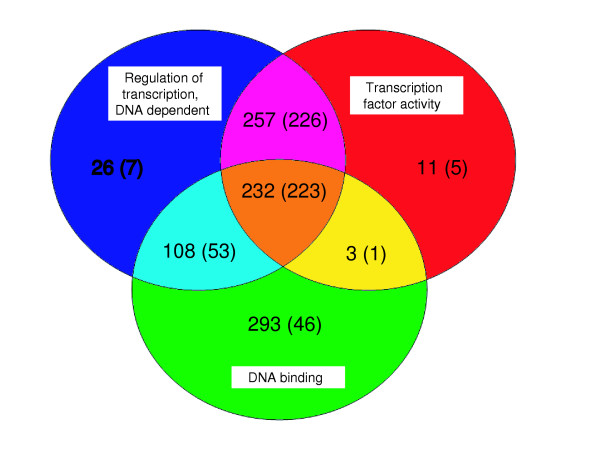
Venn diagram presenting the results of the GO term-based bioinformatic identification of putative TFs in WormBase 140. GO terms are indicated in each Venn diagram set. Numbers between parentheses represent the number of putative TFs retained in wTF2.0 after manual curation or DNA binding domain identification using InterPro v. 10.0.

To identify a comprehensive compendium of predicted worm TFs, we first interrogated WormBase version 140 (WS140)[6] for proteins that possess domains annotated with one of the following Gene Ontology (GO) terms: 'regulation of transcription, DNA-dependent', 'transcription factor activity', and 'DNA binding'. (WS140 is the most recent reference release of WormBase that is permanently accessible.) We identified a total of 930 proteins (Figure 2, Additional data file 1). Of these, 232 were identified by all three GO terms, 368 by two GO terms and 330 by only one GO term (Figure 3). We observed that this collection of proteins not only contains predicted regulatory TFs, but also proteins that function in other nuclear processes (for example, DNA replication and repair). Moreover, it contains numerous false positive predictions (for example, small GTP-binding proteins). We removed both types of false positives (Figure 2, Additional data file 2). Next, we examined each of the remaining proteins for the presence of a predicted DNA binding domain either by visual inspection of the protein sequence (AT-hooks and C2H2 zinc-fingers), or using InterPro v10.0 (2005)[22], SMART [23] and Pfam [24] databases (Additional data file 2). We found several proteins that, upon closer inspection, do not possess a DNA binding domain despite their WormBase protein domain annotation (Additional data file 2). For instance, several proteins were found that are annotated to be a NHR TF, but that only contain a predicted ligand binding domain. However, we retained proteins that do not have a clear DNA binding domain but for which experimental evidence is available that supports their function as a TF. For example, we included SKN-1, a bZIP protein known to bind DNA in a sequence-specific manner [25]. In total, 369 proteins (40%) were removed (Figure 2a, Additional data file 2). As expected, combining all three GO terms was the most robust method for identifying predicted TFs, as 96% of these proteins were retained. The GO term DNA binding by itself was least robust as only 16% of these were retained. However, this can readily be explained by the retrieval of proteins that do bind DNA but that are not involved in transcriptional regulation.

### TF predictions: DNA binding domains

Upon examination of the remaining 561 proteins, we noticed that several well known TF families were underrepresented compared to wTF1.0, or even absent (for example, bHLH, C2H2 zinc-fingers and MADF (Mothers Against Dpp Factor)). This suggests that the predictions based on GO annotations alone suffer from a high false negative rate. To address this issue, we searched WormBase for each protein domain known to be involved in sequence specific DNA binding (Table 1). In addition, we added several TFs found by yeast one-hybrid assays (for example, TFs containing RPEL and FLYWCH domains [26] (data not shown)). We used visual inspection (C2H2 zinc-fingers and AT-hooks), InterPro, SMART and Pfam to verify these predictions and, in total, added 369 additional, putative TFs to the compendium. Finally, we added 4 proteins: 3 of which are homologs of known mammalian TFs (BAR-1, HMP-2 and WRM-1, homologs of mammalian β-catenin) and one that has been described in the literature (SDC-2 [27]). In total, amongst the 19,735 predicted protein-coding genes, we identified 934 predicted *C. elegans *TF genes (Additional data file 1). Taken together, the combination of computational queries and manual curation results in a comprehensive compendium of *C. elegans *TF-encoding genes. We refer to this compendium as wTF2.0.

### TF families

Table 1 presents wTF2.0 grouped into TF families. Interestingly, 23 TFs contain DNA binding domains from different families (Additional data file 3). Future studies will determine if and how these domains function together in DNA binding specificity and, consequently, target gene selection. Interestingly, most human orthologs of these TFs also contain multiple, distinct DNA binding domains (Additional data file 3), indicating that the occurrence of multiple DNA binding domains in a TF is not worm specific. Comparison of wTF2.0 to wTF1.0 revealed that, of 48 TF families, 23 are unique to wTF2.0 (Table 1). This is likely because for the collective predictions in wTF1.0, only the major DNA binding domains were included. In addition, some protein domains have only recently been annotated to function in DNA-binding, including SAND [28], and THAP zinc-finger [29] domains. For several of the domains unique to wTF2.0 (CP2, WH-DAC, IPT/TIG, TEA/ATTS, WT1, YL1), the genes encoding them were not actually annotated until after WormPep 20 and could, therefore, not have been included in the wTF1.0 collections.

#### wTF2.0 is a dynamic resource

wTF2.0 is the most comprehensive compendium of predicted worm TFs to date. However, the set of predicted *C. elegans *TFs will still be dynamic due to regular updating of the *C. elegans *genome annotation, for example in response to genome sequence data from related nematode species [14] and improvements in gene-prediction software. We have noted several changes in gene annotations in WormBase releases subsequent to WS140 that affect wTF2.0 (Additional data file 4). There have been additions, such as Y55F3AM.7 (created in WS146 and encoding a C2H2 zinc-finger protein), and eliminations, such as Y60A9.2 (encoding a CCCH zinc-finger protein in WS140, but designated a pseudogene since WS141). We have also noted more subtle adjustments in gene structure based on TWINSCAN [13] suggestions of different splicing patterns. For example, a modified gene structure for F22A3.5 that meant the gene product would then include a complete homeodomain was adopted in WS143, with support from *C. briggsae *genome sequence. Similar gene structure changes that would lead to intact homeodomains for F34D6.2, R04A9.5 and *ceh-31*, and an intact bHLH domain for *hlh-19 *(see comments in Additional data file 1) may yet be incorporated into WormBase. Taken together, we expect that wTF2.0 will be a dynamic resource but that a relatively small number of TFs will be removed and added over time.

### Functional TFs: splice variants

wTF2.0 is a starting point to predict the actual number of functional TF complexes. For instance, the number of active TFs is likely greater than 934 because many TF genes encode multiple proteins as a result of alternative transcripts. In addition, many TFs function as heterodimers, with subunits associating in different combinations. To date, 144 of the 934 predicted TF genes (15.4%) are known to undergo alternative splicing (Additional data file 5). On average, each spliced TF gene results in 3 different transcripts and the number of splice variants per gene ranges from 2 to 13. Some alternative transcripts do not result in the expression of a different TF protein variant. In total, 379 alternative TF protein variants are expressed from 144 genes, and the number of variant proteins per TF gene is between 2 and 10. Interestingly, 30 TF variants, corresponding to 25 TF genes, no longer contain a DNA binding domain. Rather than binding DNA and regulating target gene expression directly, these proteins may have regulatory functions to control TF activity. Taken together, alternative splicing yields 205 additional putative DNA binding TFs, bringing the total number of predicted *C. elegans *TFs to 1,139. Interestingly, Taneri and colleagues [30] observed that mouse TF genes are more likely to undergo alternative splicing than other mouse genes (62% compared to 29%). These alternatively spliced TF genes may yield functionally different TFs that may bind DNA with different specificities and affinities and, as a consequence, regulate different sets of target genes. In contrast, the percentage of *C. elegans *TF genes that undergo alternative splicing is only slightly higher than the percentage of all protein-coding genes that are alternatively spliced (15% versus 10%) [31] (this study). This observation suggests that higher percentages of TF gene splicing may contribute to increased organism complexity. Finally, it is important to note that several *C. elegans *TFs can be expressed from multiple alternative promoters (Additional data file 5). Alternative promoters are likely to drive different patterns and levels of TF production, which may contribute to the complexity of combinatorial gene expression.

### Functional TFs: dimers

**Figure 4:**
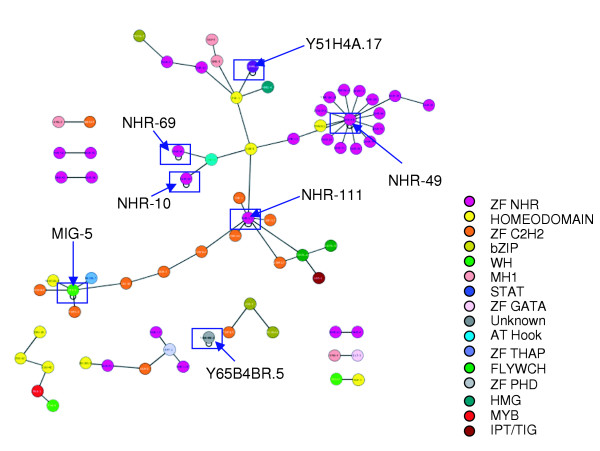
Protein-protein interaction network of worm TFs. Blue rectangles indicate homodimers. Different colors identify different TF families as indicated. Interactions were obtained from Worm Interactome version 5 (WI5) [32] and visualized using Cytoscape [59].

Several TFs, including bHLH, NHR and bZIP proteins, are known to bind DNA as either homo- or heterodimers, and the different dimer combinations that occur *in vivo *determine the actual number of TF complexes. For instance, the minimum number of TF complexes would be half the total number of TFs predicted if each TF were to exclusively dimerize with one other TF. Alternatively, the total number of functional TFs could be much larger than the number of predicted TF genes if each TF dimerizes with multiple other TFs. To start addressing this issue, we retrieved a network of TF-TF interactions that were identified in a large-scale yeast two-hybrid-based protein-protein interaction mapping study [32] (Figure 4, Additional data file 6). We found 68 putative TF-TF dimers involving 71 TFs: 35 between members of different TF families and 33 between members of the same TF family. Of these 33, 7 are putative homodimers and the remaining 26 are putative heterodimers. Interestingly, the TF dimerization network suggests that certain TFs, such as NHR-49, can function as dimerization hubs. NHR-49 is involved in the regulation of fat storage and life span [33], but it is not known if NHR-49 functions in these processes as a homodimer or in concert with other NHR TFs. It is noted that the current TF dimerization network is only a small representation of all TF dimers. This is because some TFs may only form dimers on their cognate DNA and may, therefore, not be detected by yeast two-hybrid assays; and because the current worm 'interactome' (WI5) only contains approximately 5% of all protein-protein interactions that can be detected by yeast two-hybrid assays [32]. Future systematic TF-TF protein-protein interaction mapping projects are required to determine the total complement of TF dimers. Although it is difficult to interpret interactions between TFs from different families, they could point to putative combinatorial regulation of target genes. Taken together, assuming that many TFs can function both as monomers and dimers, the number of functional TFs will likely exceed the number of predicted individual TF proteins.

#### TF families: redundancy

For the systematic mapping of transcription regulatory networks, it is important to identify redundancy between closely related TF genes. This is because redundant genes have similar, overlapping or identical biological functions and, thus, results obtained with an individual TF may be difficult to interpret. In addition, one would like to identify paralogous TFs that share extensive similarity in their DNA binding domain, because such TFs may bind similar DNA sequences and, therefore, overlapping sets of target genes. There are several well characterized examples of redundant *C. elegans *TFs, including the Fork Head genes *pes-1 *and *fkh-2 *[34], the GATA factors *med-1 *and *med-2*, and *end-1 *and *end-3 *[35], and the T-box genes *tbx-8 *and *tbx-9 *[36], and *tbx-37 *and *tbx-38 *[37]. To identify additional putative redundant or highly similar TF genes, we used ClustalX analysis to generate trees that display the level of sequence similarity within each TF family (Additional data file 7). As expected, the known redundant TFs indicated above are found on adjacent branches in these trees. Table 2 provides additional TF pairs that share extensive homology and that, therefore, may be (partially) redundant.

### Human TF orthologs

Next, we identified putative human orthologs for each worm TF, based on reciprocal best BLAST hits [38] (Additional data file 8). First, we identified members of each TF family in humans. Subsequently, we determined the number of ortholog pairs per TF family (Table 1). We found that some TF families are expanded in humans. For example, the CP2, C2HC and helix-turn-helix (HTH) TF families are represented by only one or two proteins in the worm each having a human ortholog. However, the human families are expanded six-fold. Conversely, other TF families are expanded in the worm, compared to human. As reported previously [39], the NHR family is expanded in worms and contains 274 predicted members (versus 43 in humans). Interestingly, the MADF family, which is composed of nine proteins in the worm, is not found in humans.

Putative TF orthologs may comprise a valuable tool to annotate TF function in either worm or human systems. For instance, although for most worm TFs the binding site is completely unknown [40], consensus DNA binding sequences are available for many human TFs and are collected in the Transfac database [41]. DNA binding domains evolve slower than other protein sequences [42] and, as a consequence, orthologous TFs recognize similar DNA sequences [43]. Therefore, DNA binding specificities of human TFs may be helpful to predict the DNA binding specificities of orthologous *C. elegans *TFs and *vice versa*. In the future, orthology of TFs will be invaluable in the study of the evolution of transcription regulatory networks.

### wTF2.0: a tool for the creation of TF-ORF resources

**Figure 5:**
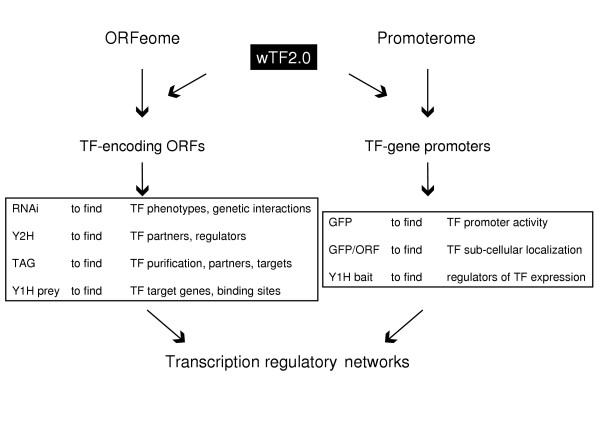
wTF2.0 can be used to create clone resources that can be used to study the transcription regulatory networks controlling metazoan gene expression. TAG, epitope or purification tag; Y1H, yeast one-hybrid; Y2H, yeast two-hybrid.

wTF2.0 provides a starting point for the creation of physical clone resources that can be used to systematically map transcription regulatory networks. TF-ORFs can be obtained from the ORFeome resource and efficiently subcloned by a Gateway cloning reaction into various different Destination vectors [44,45] (Figure 5). To date, the *C. elegans *ORFeome consists of approximately 13,000 full-length ORFs, which is approximately 66% of all predicted ORFs. We searched worfdb, the ORFeome database [46], and found 652 predicted TF-encoding ORFs (70%). These TF-ORFs can be used to map transcription regulatory networks in different ways. First, they can be cloned into yeast one-hybrid prey vectors to detect physical interactions with their target genes [26]. For instance, TF-ORFs have been pooled to create a TF mini-library that can be used in high-throughput yeast one-hybrid assays [26]. Second, they can be transferred to yeast two-hybrid or 'TAG' vectors for the identification of protein-protein interactions [47,48]. This will be important to further identify functional TF complexes and to understand how TF function is regulated. In addition, TAG vectors may be useful to create transgenic worm strains that can be used in chromatin-immunoprecipitation experiments to identify TF target genes *in vivo*. Finally, TF-ORFs can be subcloned into an RNA interference (RNAi) vector for the analysis of loss-of-function phenotypes or for the identification of genetic interactions [49-51]. Phenotypic analyses of TFs will be important for the analysis and interpretation of transcription regulatory networks.

### wTF2.0: a tool for the creation of TF gene promoter resources

To date, the Promoterome [11] contains approximately 6,500 promoters (33%), including 279 (30%) TF-promoters. TF-promoters can be fused to green fluorescent protein (GFP) in two configurations. TF-promoters can be fused directly to GFP in what are referred to as 'transcriptional fusions' and the resulting promoter::GFP constructs can be used to create transgenic *C. elegans *strains in which promoter activity can be examined by light microscopy [52]. Such lines can also be used to examine the effects on GFP expression as the result of a knockdown in regulatory TF levels by RNAi [53]. Alternatively, TF-promoters and corresponding TF-ORFs can be cloned together with GFP by multisite Gateway cloning [54] to create 'translational fusions' with GFP. The resulting promoter::ORF::GFP constructs are used to create transgenic lines in which both TF-promoter activity and TF subcellular localization can be examined [11]. Finally, TF-promoters can be cloned into yeast one-reporter vectors to identify other TFs that can physically associate with these promoters and that may contribute to TF promoter activity [26]. Such interactions are important to delineate regulatory cascades, important building blocks in transcription regulatory networks [3].

## Conclusions

We have compiled wTF2.0, a comprehensive compendium of putative *C. elegans *TFs, using both computational queries and manual curation. Combining wTF2.0 with different physical TF clone resources provides the first step toward the systematic dissection of *C. elegans *transcription regulatory networks.

## Materials and methods

### Prediction of C. elegans TF-encoding genes

WormPep 140 (WS140) (22,420 proteins, 19,735 genes) was searched. Proteins that have no apparent function in transcription regulation (for example, proteins involved in DNA repair and replication, chromatin remodeling, kinases) were removed. In addition, we removed general TFs (Additional data file 2). To identify TFs missed by the GO search, we searched WormPep 140 using individual DNA binding domains (Table 1). Next, we computationally (using SMART, Pfam or InterPro) or manually inspected each protein sequence for the presence of a DNA binding domain (Additional data file 2). For C2H2 zinc-fingers, we only considered proteins that contain fingers with the following configuration: C-X_2-5_-C-X_9_-H-X_3-5_-H [55]. However, we did include two proteins (LIR-1 and TLP-1) that do not have a canonical C2H2 zinc-finger, because they were found multiple times in high-throughput yeast one-hybrid assays (data not shown). For AT-hook predictions, we used the definition as described [56]. The numbers of human genes encoding each TF DNA binding domain were found by searching for the appropriate InterPro domain accession number using the Ensembl Human database.

### Identification of candidate redundant TFs

For each TF family with at least five members, an alignment was created using the multiple alignment mode of ClustalX v. 1.83 [57] under default settings, including the Gonnet series of protein matrices. For TF genes encoding multiple isoforms, if the DNA binding domain was identical in all isoforms, the largest isoform was used. Isoforms containing different DNA binding domains were included separately. Unrooted trees were then generated from these alignments using ClustalX v. 1.83 using the Neighbor-Joining method, and visualized using the phylogram output of TREEVIEW PPC v1.6.6 [58] (Additional data file 7). Although the analysis was not of sufficient depth for these trees to represent real evolutionary relationships amongst the deeper branches, these trees do accurately reflect close relationships between *C. elegans *TFs, with candidate redundant genes occurring on adjacent short branches.

### TF splice variants and alternative TF promoters

To identify TF splice variants, the coding sequences of each TF were retrieved from WS140 using the batch gene tool. Each splice variant was then manually examined for the presence of a DNA-binding domain and/or an alternative promoter.

### TF dimers

TF dimers were obtained from worm interactome version 5 (WI5) [32]. TF-TF interactions were modeled into a protein interaction network using the Cytoscape software package [59]. In this network, nodes correspond to interactors and edges (that is, links between nodes) represent protein-protein interactions.

### TF orthologs

For each TF, the best human blastp hit was retrieved from WormBase. The Ensembl ID of each retrieved human protein was then used to extract the best *C. elegans *blastp hit and the corresponding percentage protein sequence identity using the data-mining tool BioMart. For the 44 TFs that did not yield a hit individual blast searches were performed. For 26 *C. elegans *TFs still no human homolog could be identified. Reciprocal best blast hits were considered putative orthologs and are highlighted in bold in Additional data file 8.

## Additional data files

The following additional data are available with the online version of this paper. Additional file 1 is a table listing the collection of predicted *C. elegans *transcription factors referred to as wTF2.0. Additional data file 2 is a table providing an overview of manually curated genes that were left out of wTF2.0. Additional data file 3 is a table listing wTF2.0 TFs that contain two distinct DNA binding domains. Additional data file 4 is a table showing possible additions to wTF2.0. Additional data file 5 is a table showing alternative splice forms and promoters. Additional data file 6 is a table that provides an overview of protein interactions involving wTF2.0 TFs. Additional data file 7 is a figure showing phylogenetic trees of worm TF families. Additional data file 8 is a table listing putative human wTF2.0 TF homologs and orthologs.

## Supplementary Material

Additional data file 1 wTF2.0: a collection of predicted *C. elegans *transcription factors Click here for file

Additional data file 2 Overview of manually curated genes that were left out of wTF2.0 Click here for file

Additional data file 3 wTF2.0 TFs that contain two distinct DNA binding domains Click here for file

Additional data file 4 Possible additions to wTF2.0 Click here for file

Additional data file 5 Alternative splice forms and promoters Click here for file

Additional data file 6 Overview of protein interactions involving wTF2.0 TFs Click here for file

Additional data file 7 Phylogenetic trees of worm TF families Click here for file

Additional data file 8 Putative human wTF2.0 TF homologs and orthologs Click here for file

## Figures and Tables

**Table 1:** Comparison of wTF2.0 versus wTF1.0

DNA binding domain	Description	wTF2.0			wTF1.0			
		
		WP140 22,420	Family members in humans	Ortholog pairs	Clarke 1998 WP14 14,655 [16]	Ruvkun 1998 WP15 15,558 [15]	Rubin 2000 WP18 18,576	Reichaman 2000 WP20 19,101 [18]
AP-2	Activator protein-2	4	5	0	-	-	-	4
ARID/BRIGHT	AT-rich interaction domain	4	9	2	-	-	-	4
AT HOOK		31	28	5	-	-	-	-
BHLH	basic region helix loop helix	42	103	22	-	24	-	25
BZIP	basic region leucine zipper	32	57	11	-	18	18	25
CBF	CCAAT-binding factor	9	12	7	-	-	-	-
COLD BOX		5	11	2	-	-	-	-
CP2		1	6	1	-	-	-	-
HD	Homeodomain	99	167	36	-	83	88	84
HMG	High mobility group	16	59	12	-	-	-	15
HTH	Helix turn helix	2	12	2	-	-	-	-
IPT/TIG	Ig-like, plexins, TFs	3	8	2	-	-	-	-
MADF	Mothers against Dpp factor	9	0	0	-	-	-	-
MADS box	MCM1/AG/DEF/SRF	2	5	2	-	-	-	2
MH1	MAD homology 1	7	12	1	-	-	-	-
MYB		19	21	8	-	-	16	3
p53		3	3	1	-	-	-	0
PD-FULL	Paired domain	5	9	1*	-	5	-	-
PD-NPAX	Paired domain	4	0	0	-	-	-	-
PD-CPAX	Paired domain	1	0	0	-	-	-	-
PD-UNDEFINED	Paired domain	-	-	-	-	-	11^†^	10^†^
RPEL		1	3	1	-	-	-	-
RUNT		1	6	1	-	-	-	1
SAND	Sp100, AIRE-1, NucP41/75, DEAF-1	4	8	1*	-	-	-	-
STAT	Signal transducers and activators of transcription	2	7	0	-	-	-	1
T-BOX		21	17	2	-	17	-	21
TEA/ATTS	Transcriptional enhancer activator	1	4	1	-	-	-	-
TSC-22/DIP/BUN		3	4	0	-	-	-	1
WH-DAC	Dachshund	1	2	1	-	-	-	-
WH-ETS	Erythroblast transformation specific	10	15	5	-	10	-	10
WH-FH	Fork head	18	41	4	-	15	19	15
WH-HSF	Heat shock factor	2	8	1	-	-	-	1
WH-RFX	X-box binding regulatory factor	1	3	0	-	-	-	1
WH-TDP	TF E2F dimerisation partner	4	11	2	-	-	-	4
WH-UNDEFINED	Winged helix	4	0	0	-	-	-	-
WT1	Wilms tumor1	1	17	0	-	-	-	-
YL1		1	1	1	-	-	-	-
ZF-A20	Zinc finger, A20-type	2	6	2	-	-	-	-
ZF-BED	BEAF/DREF-like ZF	6	4	1	-	-	-	-
ZF-C2H2		211	391	35	117	-	138	139
ZF-C2HC		1	6	1	-	-	-	-
ZF-CCCH		32	50	8	20	-	-	15
ZF-DHHC		15	21	7	-	-	13	-
ZF-DM	Dsx and Mab-3-like ZF	11	7	0	8	-	-	9
ZF-FLYWCH		4	1	0	-	-	-	-
ZF-GATA		14	7	3	9	-	-	9
ZF-MIZ	Msx interacting ZF	2	6	1	-	-	-	-
ZF-NF-X1	Nuclear factor	2	3	2	-	-	-	-
ZF-NHR/C4	Nuclear hormone receptor	274	43	6	233	235	224	252
ZF-THAP		5	12	2	-	-	-	-
UNKNOWN		5	0	2	-	-	-	-
TOTAL		957^‡^	1,231	203	387	407	527	652

This table shows the number of genes encoding each type of domain. Genes encoding multiple domains of the same type are counted only once. Dashes indicate the domain was not investigated. *These genes encode two distinct domains: PD and HD; SAND and AT hook. ^†^Without access to the complete Rubin and Reichmann lists, we are unable to classify their PD family members. ^‡^Twenty-three genes in wTF2.0 encode two different types of domain.

**Table 2:** Candidate redundant worm TF pairs

DNA binding domain	TF 1	TF 2	E-value	% Identity
T-BOX	Y59E9AR.5	TBX-30	E-168	100
T-BOX	TBX-39	TBX-40	E-170	98.9
ZF CCCH	C35D6.4	F38C2.7	E-98	99
COLD BOX	CEY-2	CEY-3	E-96	99.6
ZF C2H2	F47H4.1	Y6G8.3	E-109	99.6
ZF C2H2	H16D19.3	T07D10.3	E-112	58.6
ZF C2H2	LSY-2	LSL-1	E-74	81.1
ZF C2H2	T07G12.10	T07G12.11	E-194	98.8
bHLH	HLH-25	HLH-27	E-139	95.6
bHLH	HLH-28	HLH-29	E-124	99.6
HMG	HMG-3	HMG-4	E-297	99.9
MYB	T10E7.11	T07F8.4	E-132	99.6
PAIRED DOMAIN	EGL-38	PAX-2	E-132	92.4
ZF - PHD	F15E6.1	Y51H4A.12	0	98
ZF - PHD	LET-418	CHD-3	0	99.7
Fork Head	FKH-3	FKH-4	E-199	99.7

E-values and % identity values were obtained via pairwise blastp BLAST. See Table 1 for DNA binding domain abbreviations.

## References

[B1] Levine M, Tjian R (2003). Transcription regulation and animal diversity.. Nature.

[B2] Lee TI, Young RA (2000). Transcription of eukaryotic protein-coding genes.. Annu Rev Genet.

[B3] Lee TI, Rinaldi NJ, Robert F, Odom DT, Bar-Joseph Z, Gerber GK, Hannett NM, Harbison CT, Thompson CM, Simon I (2002). Transcriptional regulatory networks in *Saccharomyces cerevisiae*.. Science.

[B4] Davidson EH, Rast JP, Oliveri P, Ransick A, Calestani C, Yuh C-H, Minokawa T, Amore G, Hinman V, Arenas-Mena C (2002). A genomic regulatory network for development.. Science.

[B5] Luscombe NM, Madan Babu M, Yu H, Snyder M, Teichmann SA, Gerstein M (2004). Genomic analysis of regulatory network dynamics reveals large topological changes.. Nature.

[B6] Chen N, Harris TW, Atoshechkin I, Bastiani C, Bieri T, Blasiar D, Bradnam K, Canaran P, Chan J, Chen CK (2005). WormBase: a comprehensive data resource for *Caenorhabditis *biology and genomics.. Nucleic Acids Res.

[B7] Hill AA, Hunter CP, Tsung BT, Tucker-Kellogg G, Brown EL (2000). Genomic analysis of gene expression in *C. elegans*.. Science.

[B8] Kim SK, Lund J, Kiraly M, Duke K, Jiang M, Stuart JM, Eizinger A, Wylie BN, Davidson GS (2001). A gene expression map for *Caenorhabditis elegans*.. Science.

[B9] Roy PJ, Stuart JM, Lund J, Kim SK (2002). Chromosomal clustering of muscle-expressed genes in *Caenorhabditis elegans*.. Nature.

[B10] Reboul J, Vaglio P, Rual JF, Lamesch P, Martinez M, Armstrong CM, Li S, Jacotot L, Bertin N, Janky R (2003). *C. elegans *ORFeome version 1.1: experimental verification of the genome annotation and resource for proteome-scale protein expression.. Nat Genet.

[B11] Dupuy D, Li Q, Deplancke B, Boxem M, Hao T, Lamesch P, Sequerra R, Bosak S, Doucette-Stam L, Hope IA (2004). A first version of the *Caenorhabditis elegans *promoterome.. Genome Res.

[B12] The *C.elegans* Sequencing Consortium (1998). Genome sequence of the nematode *C. elegans *: a platform for investigating biology.. Science.

[B13] Wei C, Lamesch P, Arumugam M, Rosenberg J, Hu P, Vidal M, Brent MR (2005). Closing in on the *C. elegans *ORFeome by cloning TWINSCAN predictions.. Genome Res.

[B14] Stein LD, Bao Z, Blasiar D, Blumenthal T, Brent MR, Chen N, Chinwalla A, Clarke L, Clee C, Coghlan A (2003). The genome sequence of *Caenorhabditis briggsae *: A platform for comparative genomics.. PLoS Biol.

[B15] Ruvkun G, Hobert O (1998). The taxonomy of developmental control in *Caenorhabditis elegans*.. Science.

[B16] Clarke ND, Berg JM (1998). Zinc fingers in *Caenorhabditis elegans *: finding families and probing pathways.. Science.

[B17] Rubin GM, Yandeu MD, Wortman JR, Gabor Miklas GL, Nelson CR, Hariharan IK, Fortini ME, Li PW, Apweiler R, Fleischmann W (2000). Comparative genomics of the eukaryotes.. Science.

[B18] Riechmann JL, Heard J, Martin G, Reuber L, Jiang C, Keddie J, Adam L, Pineda O, Ratcliffe OJ, Samaha RR (2000). *Arabidopsis* transcription factors: genome-wide comparative analysis among eukaryotes.. Science.

[B19] Ledent V, Paquet O, Vervoort M (2002). Phylogenetic analysis of the human basic helix-loop-helix proteins.. Genome Biol.

[B20] Burglin TR, Cassata G (2002). Loss and gain of domains during evolution of cut superclass homeobox genes.. Int J Dev Biol.

[B21] Volff JN, Zarkower D, Bardwell VJ, Schartl M (2003). Evolutionary dynamics of the DM domain gene family in metazoans.. J Mol Evol.

[B22] Mulder NJ, Apweiler R, Attwood TK, Bairoch A, Bateman A, Binns D, Bradley P, Bork P, Bucher P, Cerutti L (2005). InterPro, progress and status in 2005.. Nucleic Acdis Res.

[B23] Letunic I, Copley RR, Schmidt S, Ciccarelli FD, Doerks T, Schultz J, Ponting CP, Bork P (2004). SMART 4.0: towards genomic data integration.. Nucleic Acids Res.

[B24] Sonnhammer EL, Eddy SR, Durbin R (1997). Pfam: a comprehensive database of protein domain families based on seed alignments.. Proteins.

[B25] Blackwell TK, Bowerman B, Priess JR, Weintraub H (1994). Formation of a monomeric DNA binding domain by Skn-1 bZIP and homeodomain elements.. Science.

[B26] Deplancke B, Dupuy D, Vidal M, Walhout AJM (2004). A Gateway-compatible yeast one-hybrid system.. Genome Res.

[B27] Chu DS, Dawes HE, Lieb JD, Chan RC, Kuo AF, Meyer BJ (2002). A molecular link between gene-specific and chromosome-wide transcriptional repression.. Genes Dev.

[B28] Bottomley MJ, Collard MW, Huggenvik JI, Liu Z, Gibson TJ, Sattler M (2001). The SAND domain structure defines a novel DNA-binding fold in transcriptional regulation.. Nat Struct Biol.

[B29] Clouaire T, Roussigne M, Ecochard V, Mathe C, Amalric F, Girard JP (2005). The THAP domain of THAP1 is a large C2CH module with zinc-dependent sequence-specific DNA-binding activity.. Proc Natl Acad Sci USA.

[B30] Taneri B, Snyder B, Novoradovsky A, Gaasterland T (2004). Alternative splicing of mouse transcription factors affects their DNA-binding domain architecture and is tissue-specific.. Genome Biol.

[B31] Brett D, Pospisil H, Valcarcel J, Reich J, Bork P (2001). Alternative splicing and genome complexity.. Nat Genet.

[B32] Li S, Armstrong CM, Bertin N, Ge H, Milstein S, Boxem M, Vidalain P-O, Han J-DJ, Chesneau A, Hao T (2004). A map of the interactome network of the metazoan *C. elegans*.. Science.

[B33] Van Gilst MR, Hajivassiliou H, Jolly A, Yamamoto KR (2005). Nuclear hormone receptor NHR-49 controls fat consumption and fatty acid composition in *C. elegans*.. PLoS Biol.

[B34] Molin L, Mounsey A, Aslam S, Bauer P, Young J, James M, Sharma-Oates A, Hope IA (2000). Evolutionary conservation of redundancy between a diverged pair of forkhead transcription factor homologues.. Development.

[B35] Maduro MF, Rothman JH (2002). Making worm guts: the gene regulatory network of the *Caenorhabditis elegans *endoderm.. Dev Biol.

[B36] Pocock R, Ahringer J, Mitsch M, Maxwell S, Woollard A (2004). A regulatory network of T-box genes and the even-skipped homologue *vab-7 *controls patterning and morphogenesis in *C. elegans*.. Development.

[B37] Good K, Ciosk R, Nance J, Neves A, Hill RJ, Priess JR (2004). The T-box transcription factors TBX-37 and TBX-38 link GLP-1/Notch signaling to mesoderm induction in *C. elegans *embryos.. Development.

[B38] Stuart JM, Segal E, Koller D, Kim SK (2003). A gene-coexpression network for global discovery of conserved genetic modules.. Science.

[B39] Sluder AE, Mathews SW, Hough D, Yin VP, Maina CV (1999). The nuclear receptor superfamily has undergone extensive proliferation and diversification in nematodes.. Genome Res.

[B40] Okkema PG, Krause M, WormBook Transcriptional regulation.. The C elegans Research Community.

[B41] Wingender E, Chen X, Fricke E, Geffers R, Hehl R, Lieblich I, Krull M, Matys V, Michael H, Ohnhauser R (2001). The TRANSFAC system on gene expression regulation.. Nucleic Acids Res.

[B42] Ruvinsky I, Ruvkun G (2003). Functional tests of enhancer conservation between distantly related species.. Development.

[B43] Conlon FL, Fairclough L, Price BM, Casey ES, Smith JC (2001). Determinants of T box protein specificity.. Development.

[B44] Walhout AJM, Temple GF, Brasch MA, Hartley JL, Lorson MA, van den Heuvel S, Vidal M (2000). GATEWAY recombinational cloning: application to the cloning of large numbers of open reading frames or ORFeomes.. Methods Enzymol.

[B45] Hartley JL, Temple GF, Brasch MA (2000). DNA cloning using *in vitro* site-specific recombination.. Genome Res.

[B46] Vaglio P, Lamesch P, Reboul J, Rual JF, Martinez M, Hill D, Vidal M (2003). WorfDB: the *Caenorhabditis elegans *ORFeome Database.. Nucleic Acids Res.

[B47] Walhout AJM, Vidal M (2001). High-throughput yeast two-hybrid assays for large-scale protein interaction mapping.. Methods.

[B48] Braun P, Hu Y, Shen B, Halleck A, Koundinya M, Harlow E, LaBaer J (2002). Proteome-scale purification of human proteins from bacteria.. Proc Natl Acad Sci USA.

[B49] Fraser AG, Kamath RS, Zipperlen P, Martinez-Campos M, Sohrmann M, Ahringer J (2000). Functional genomics analysis of *C. elegans *chromosome I by systematic RNA interference.. Nature.

[B50] Rual J-F, Ceron J, Koreth J, Hao T, Nicot A-S, Hirozane-Kishikawa T, Vandenhaute J, Orkin SH, Hill DE, van den Heuvel S (2004). Toward improving *Caenorhabditis elegans *phenome mapping with an ORFeome-based RNAi library.. Genome Res.

[B51] Baugh LR, Wen JC, Hill AA, Slonim DK, Brown EL, Hunter CP (2005). Synthetic lethal analysis of *Caenorhabditis elegans *posterior embryonic patterning genes identifies conserved genetic interactions.. Genome Biol.

[B52] Hope IA, Stevens J, Garner A, Hayes J, Cheo DL, Brasch MA, Vidal M (2004). Feasibility of genome-scale construction of promoter::reporter gene fusions for expression in *Caenorhabditis elegans *using a multisite Gateway recombination system.. Genome Res.

[B53] Morley JF, Morimoto RI (2004). Regulation of longevity in *Caenorhabditis elegans *by heat shock factor and molecular chaperones.. Mol Biol Cell.

[B54] Cheo DL, Titus SA, Byrd DRN, Hartley JL, Temple GF, Brasch MA (2004). Concerted assembly and cloning of multiple DNA segments using *in vitro* site-specific recombination: functional analysis of multi-segment expression clones.. Genome Res.

[B55] Wolfe SA, Nekludova L, Pabo CO (2000). DNA recognition by Cys2His2 zinc finger proteins.. Annu Rev Biophys Biomol Struct.

[B56] Aravind L, Landsman D (1998). AT-hook motifs identified in a wide variety of DNA-binding proteins.. Nucleic Acids Res.

[B57] Thompson JD, Gibson TJ, Plewniak F, Jeanmougin F, Higgins DG (1997). The CLUSTAL_X windows interface: flexible strategies for multiple sequence alignment aided by quality analysis tools.. Nucleic Acids Res.

[B58] Page RDM (1996). TREEVIEW: an application to display phylogenetic trees on personal computers.. Computer Appl Biosci.

[B59] Shannon P, Markiel A, Ozier O, Baliga NS, Wang JT, Ramage D, Amin N, Schwikowski B, Ideker T (2003). Cytoscape: a software environment for integrated models of biomolecular interaction networks.. Genome Res.

