# Supplementary material for: A compendium of Caenhorabditis elegans regulatory transcription factors: a resource for mapping transcription regulatory networks
Source: Genome Biol. 2005 Dec 30;6(13):R110. doi: 10.1186/gb-2005-6-13-r110 (PMC1414109; doi:10.1186/gb-2005-6-13-r110)
Supplement: Additional data file 7 — Phylogenetic trees of worm TF families [file gb-2005-6-13-r110-S7.pdf]

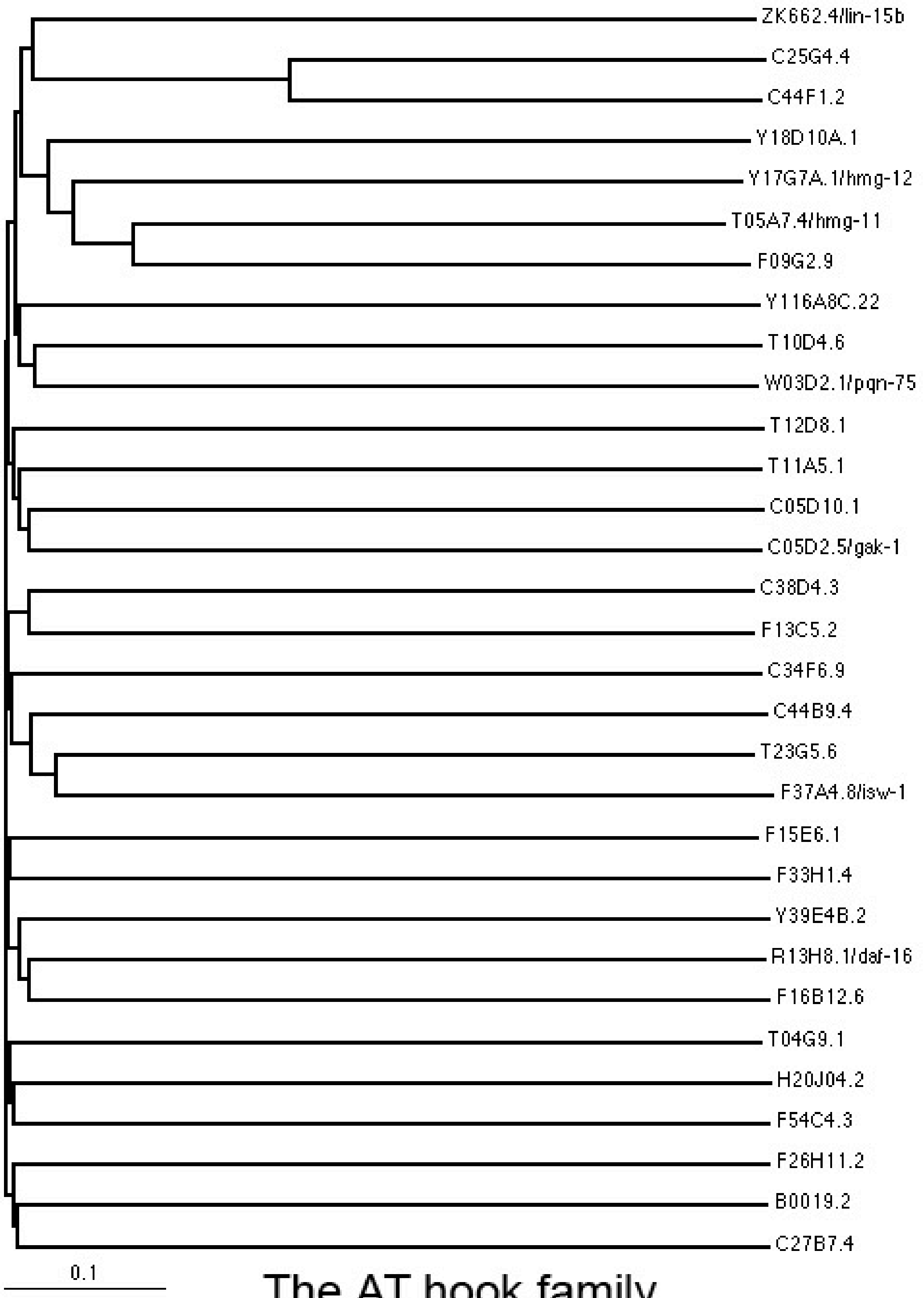

The AT hook family

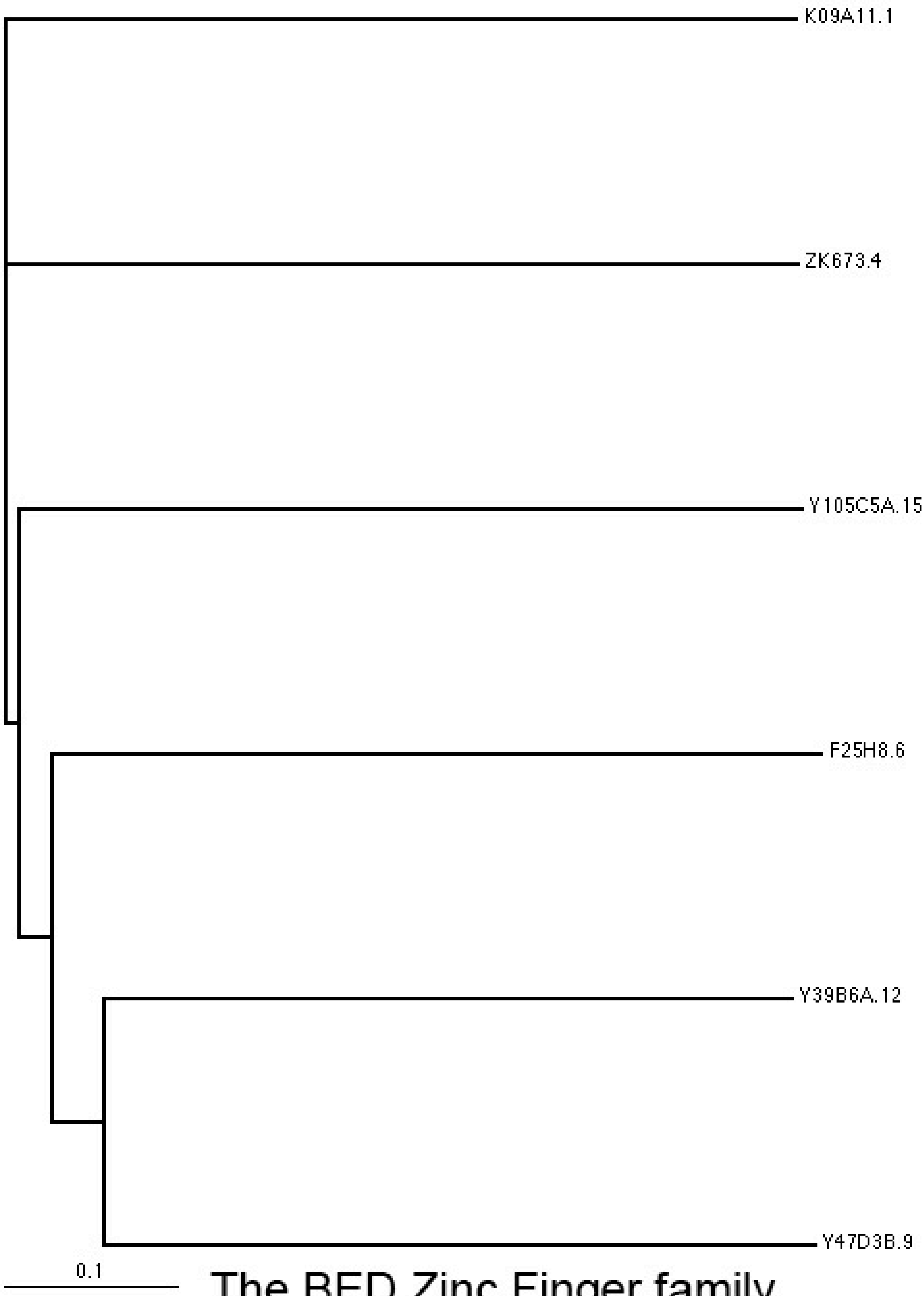

The BED Zinc Finger family

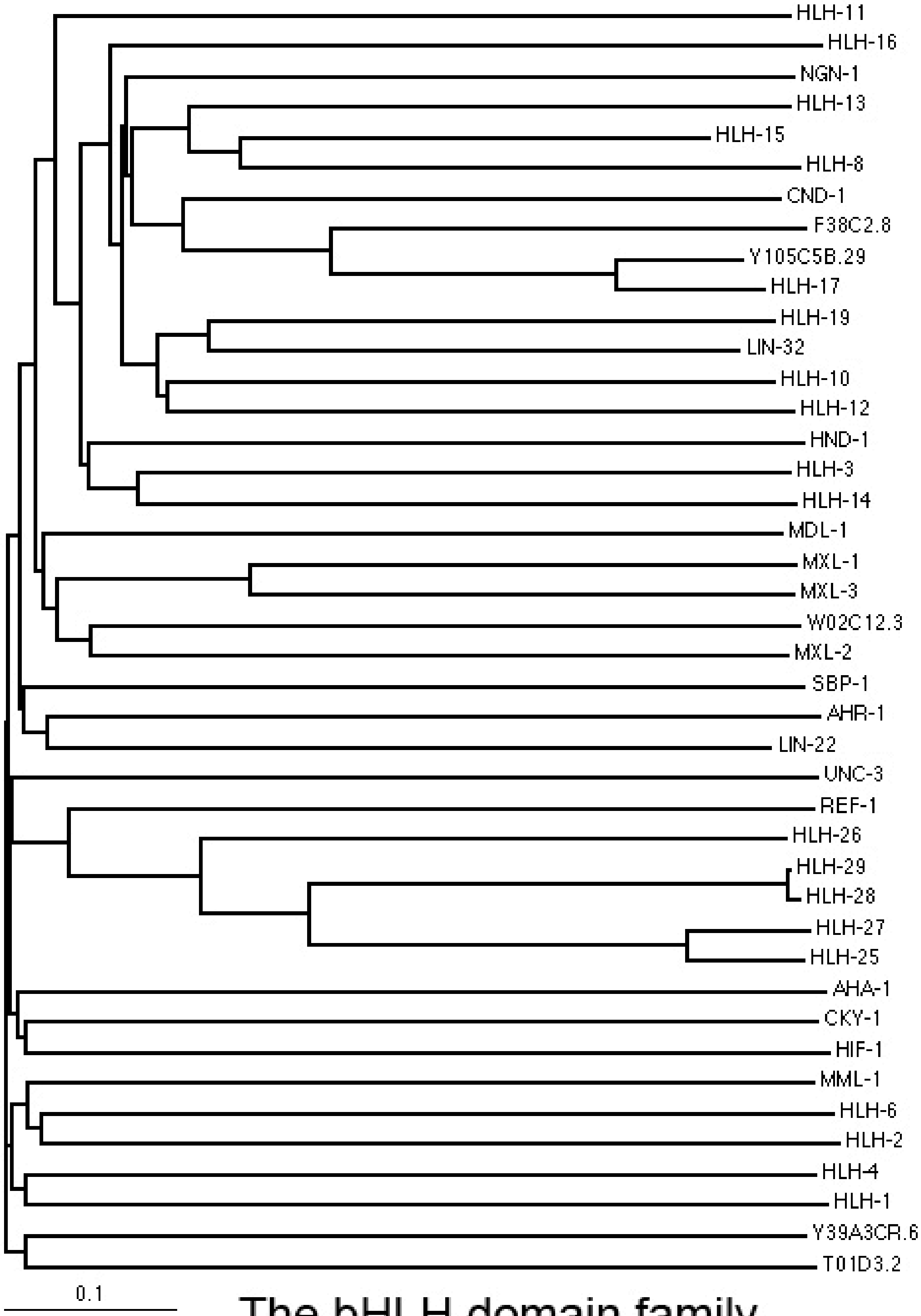

The bHLH domain family

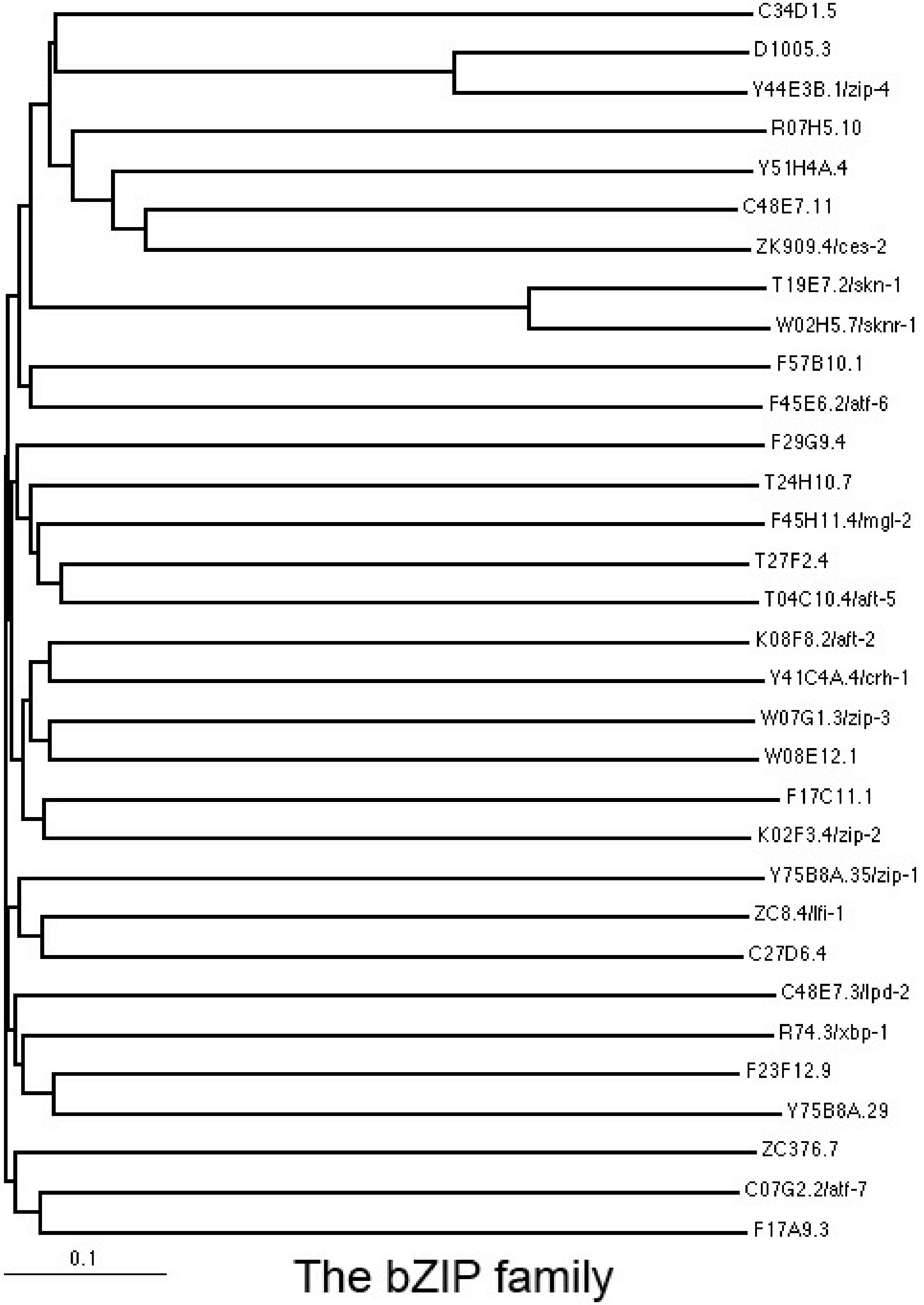

The bZIP family

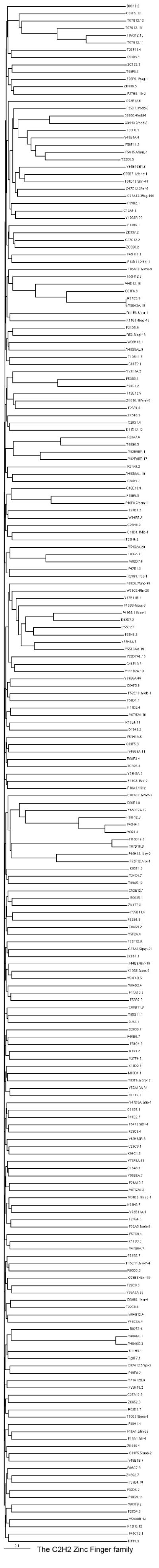

The C2H2 Zinc Finger family

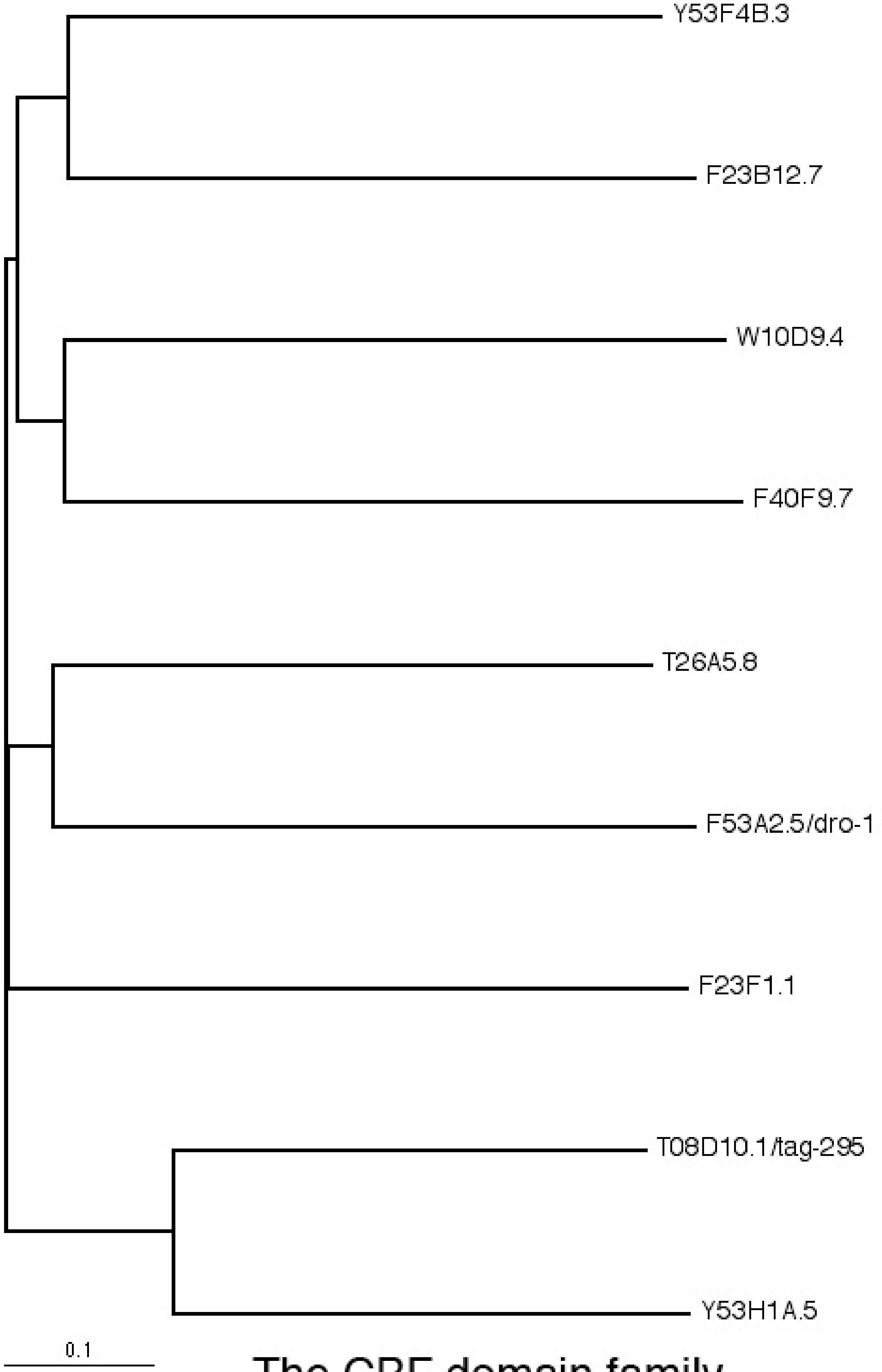

The CBF domain family

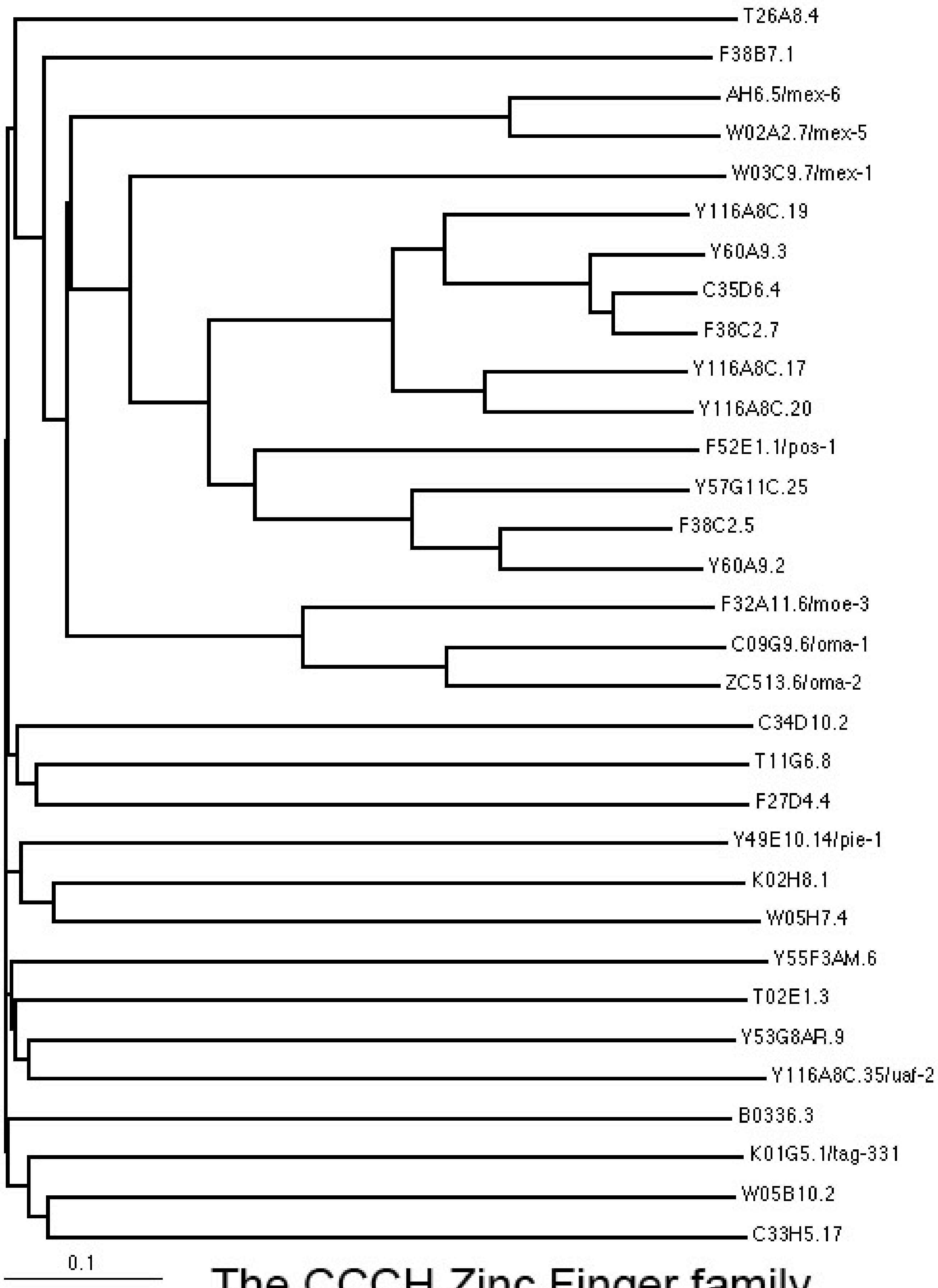

The CCCH Zinc Finger family

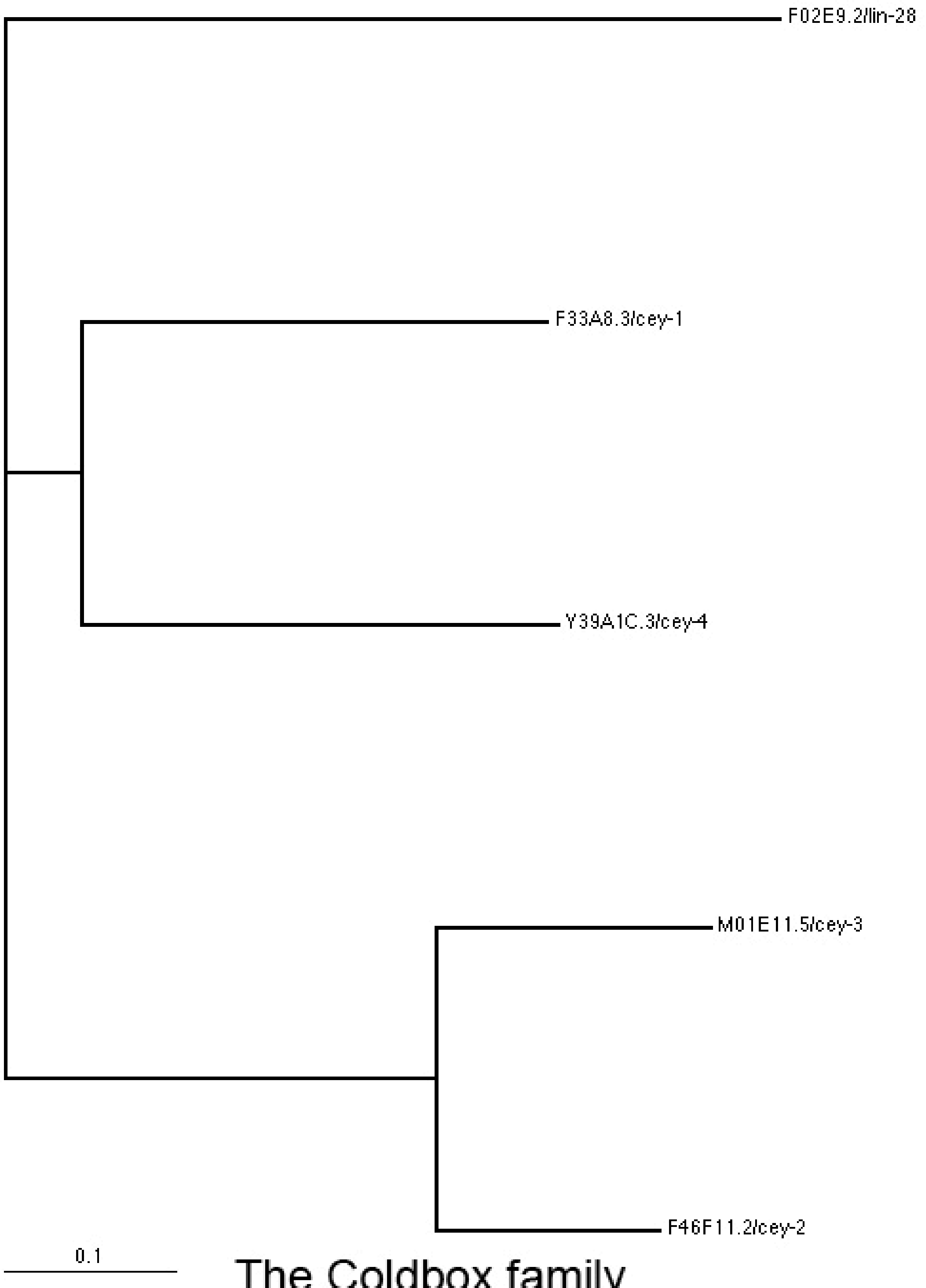

The Coldbox family

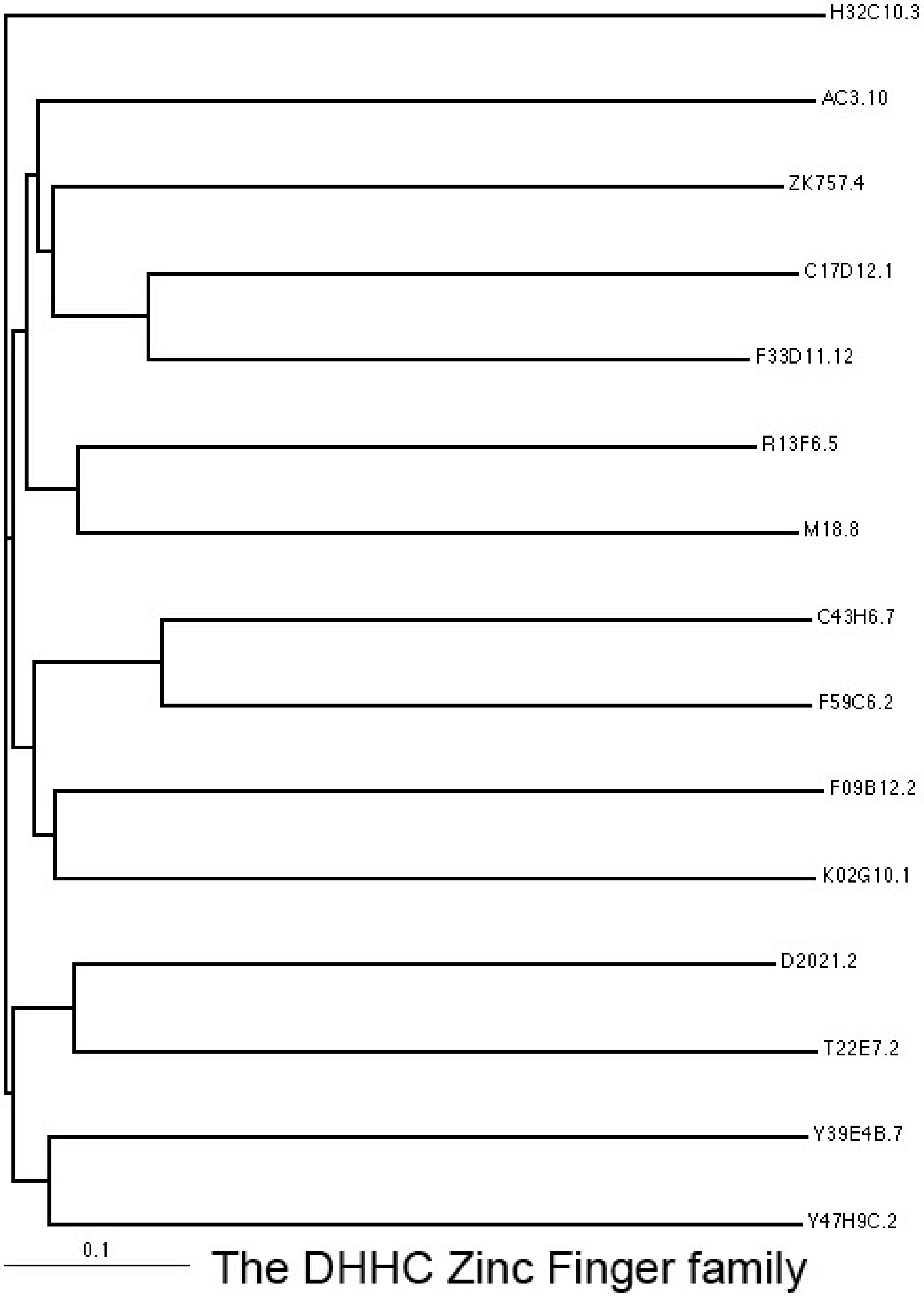

The DHHC Zinc Finger family

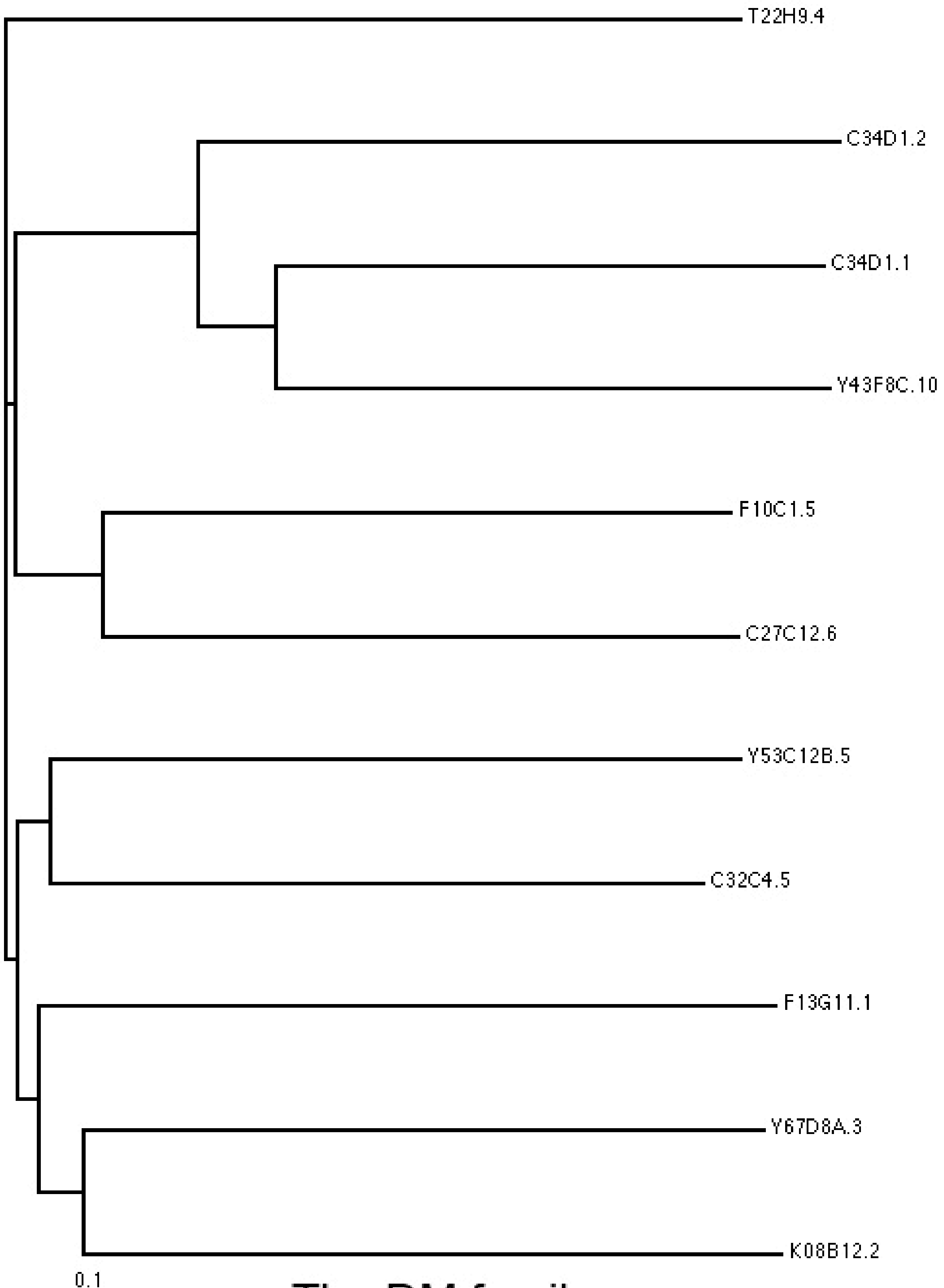

The DM family

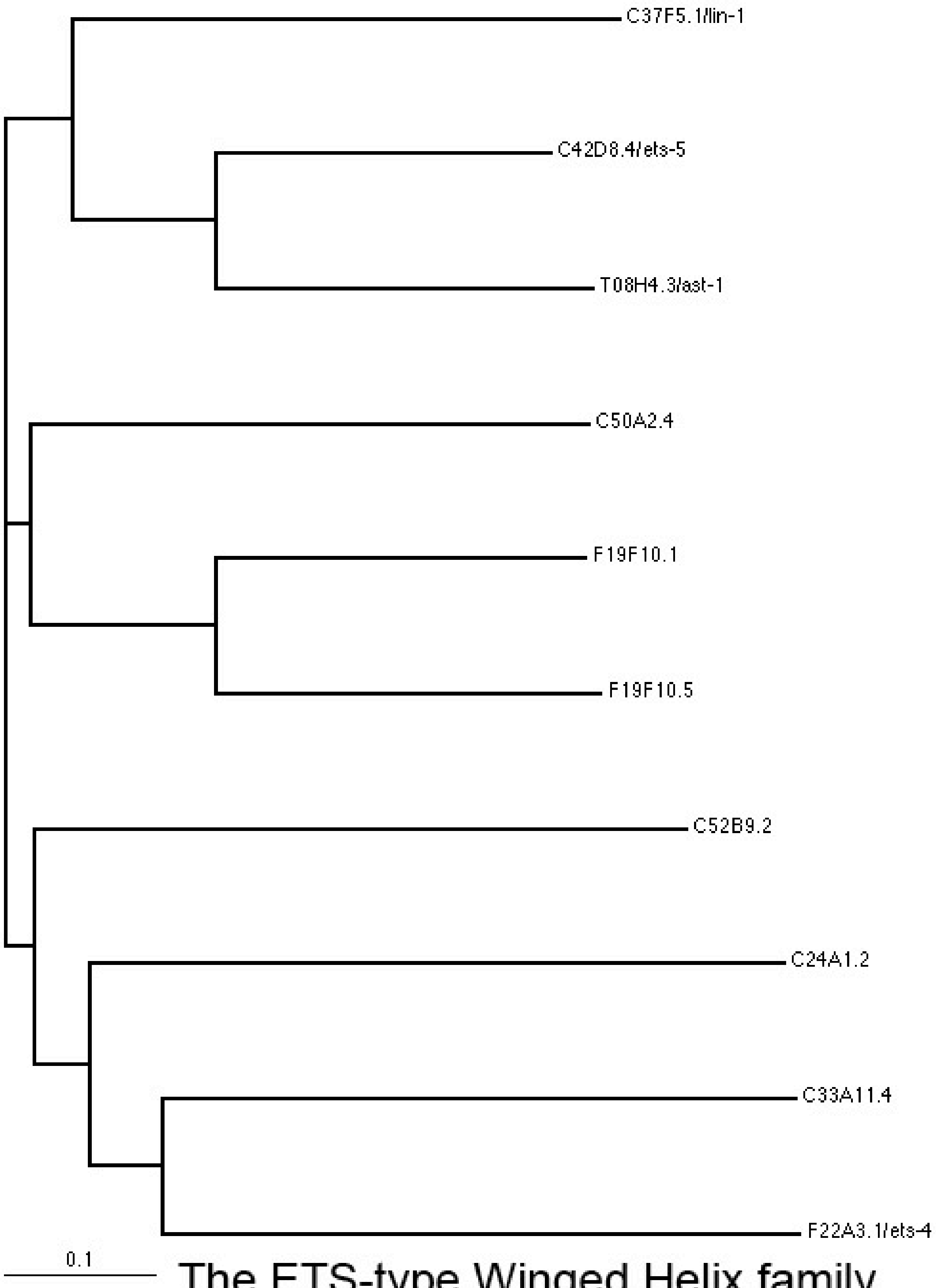

The ETS-type Winged Helix family

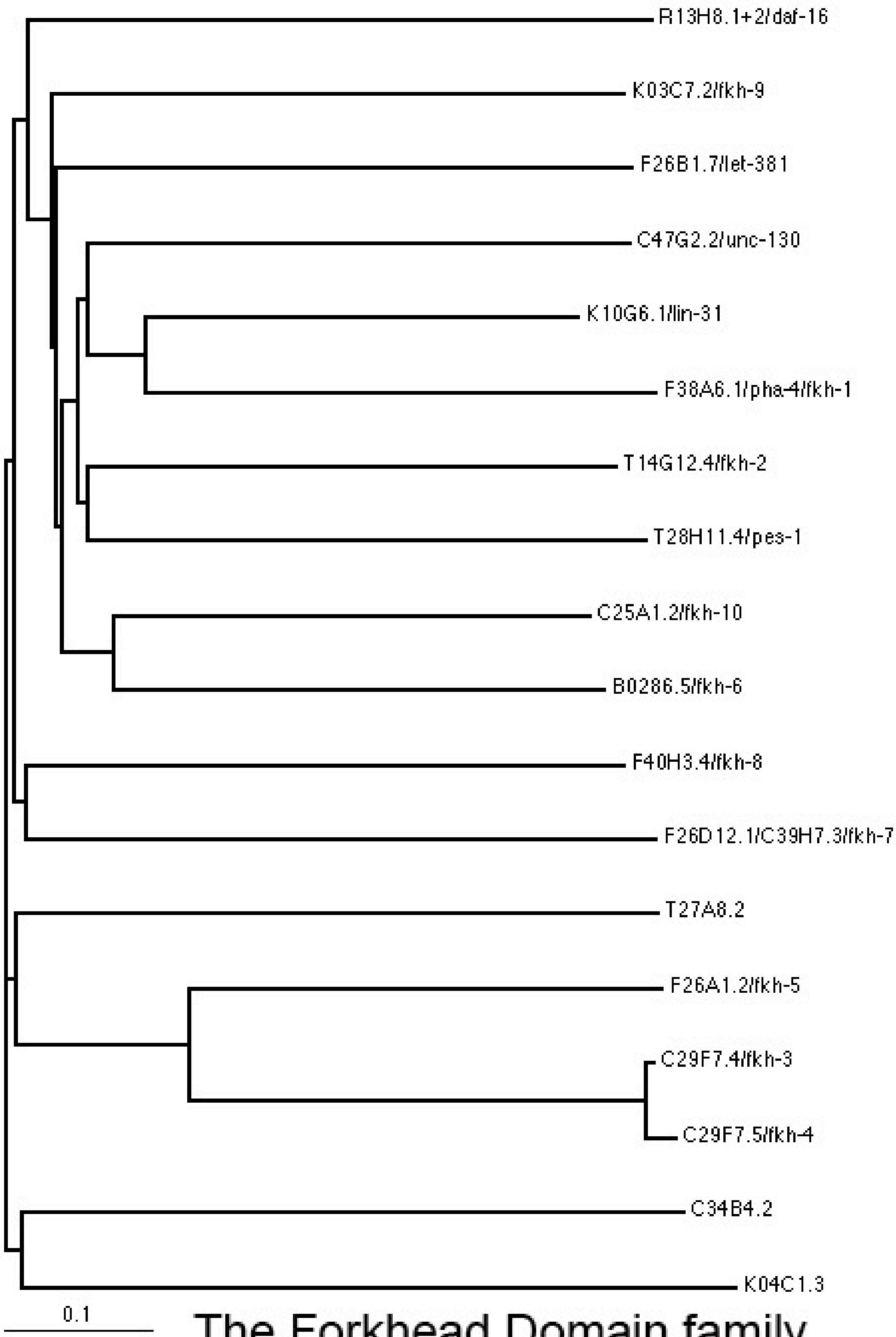

The Forkhead Domain family

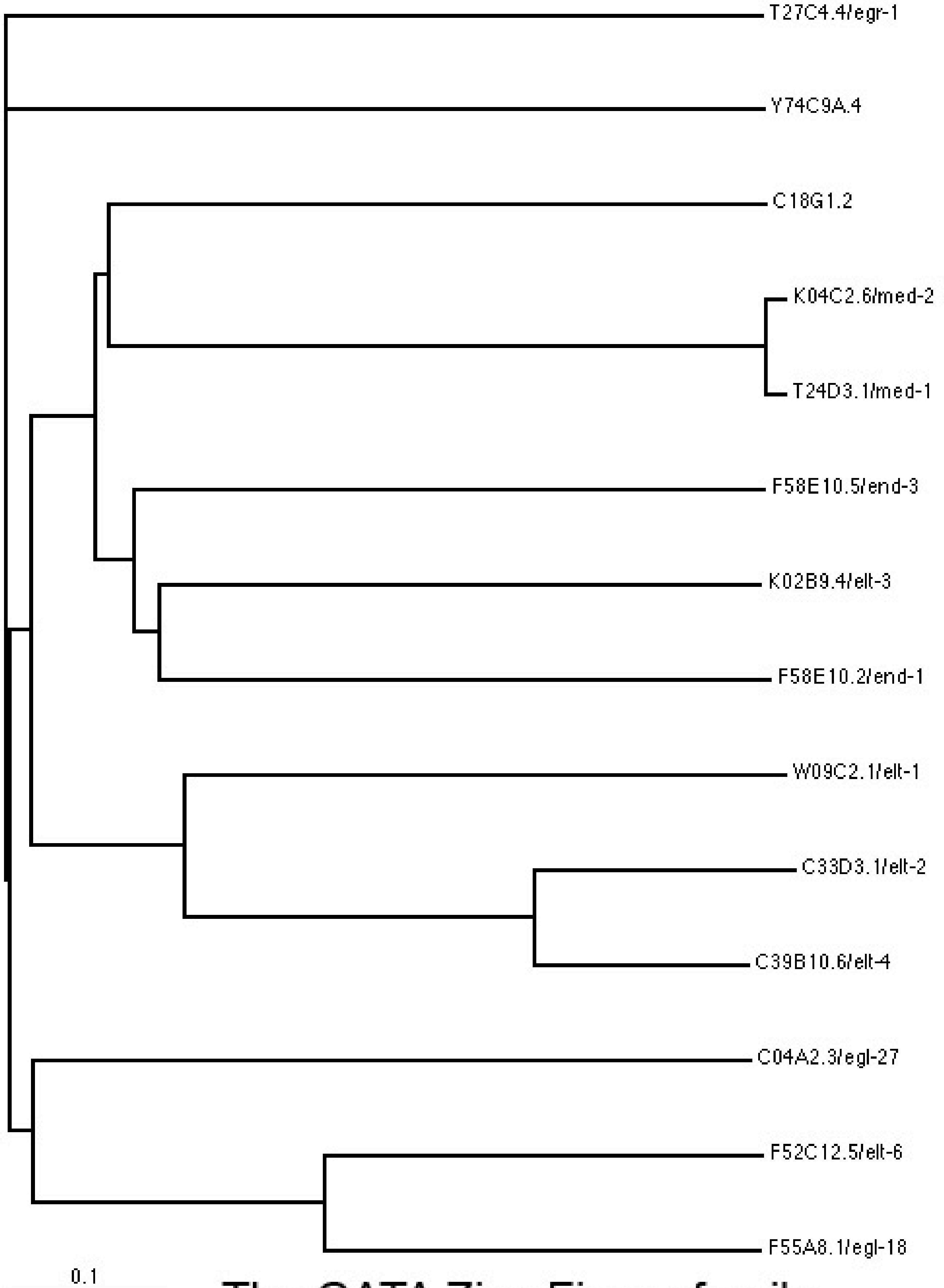

The GATA Zinc Finger family

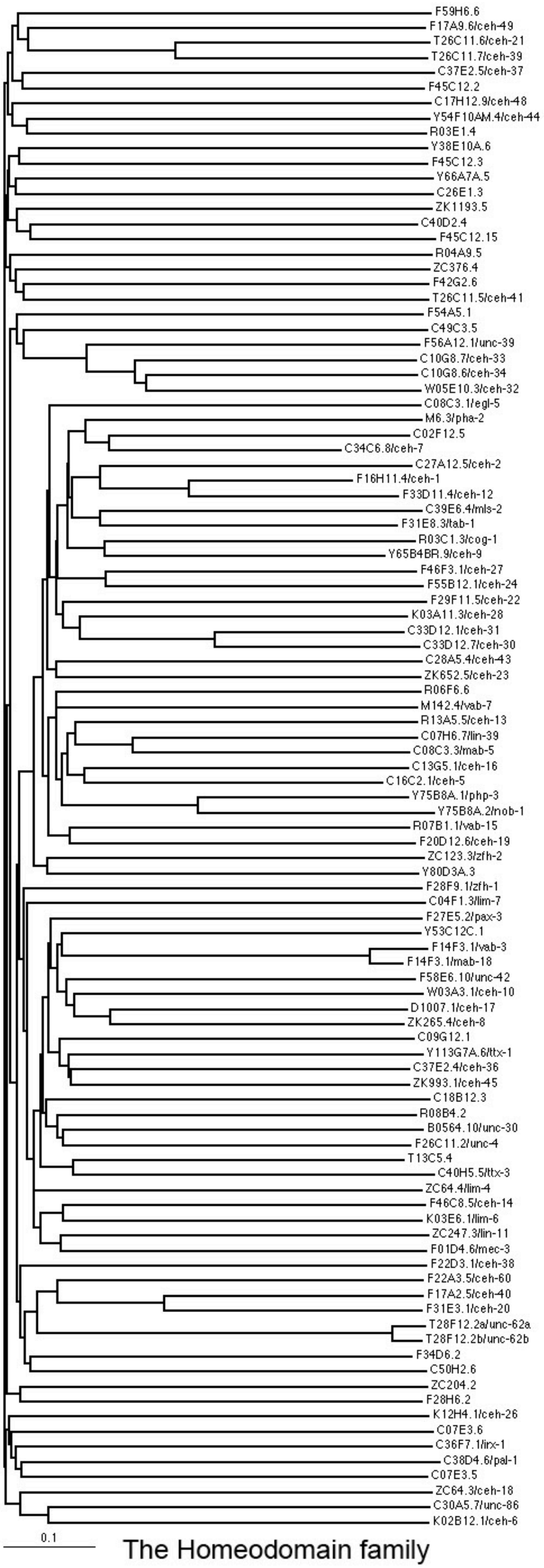

The Homeodomain family

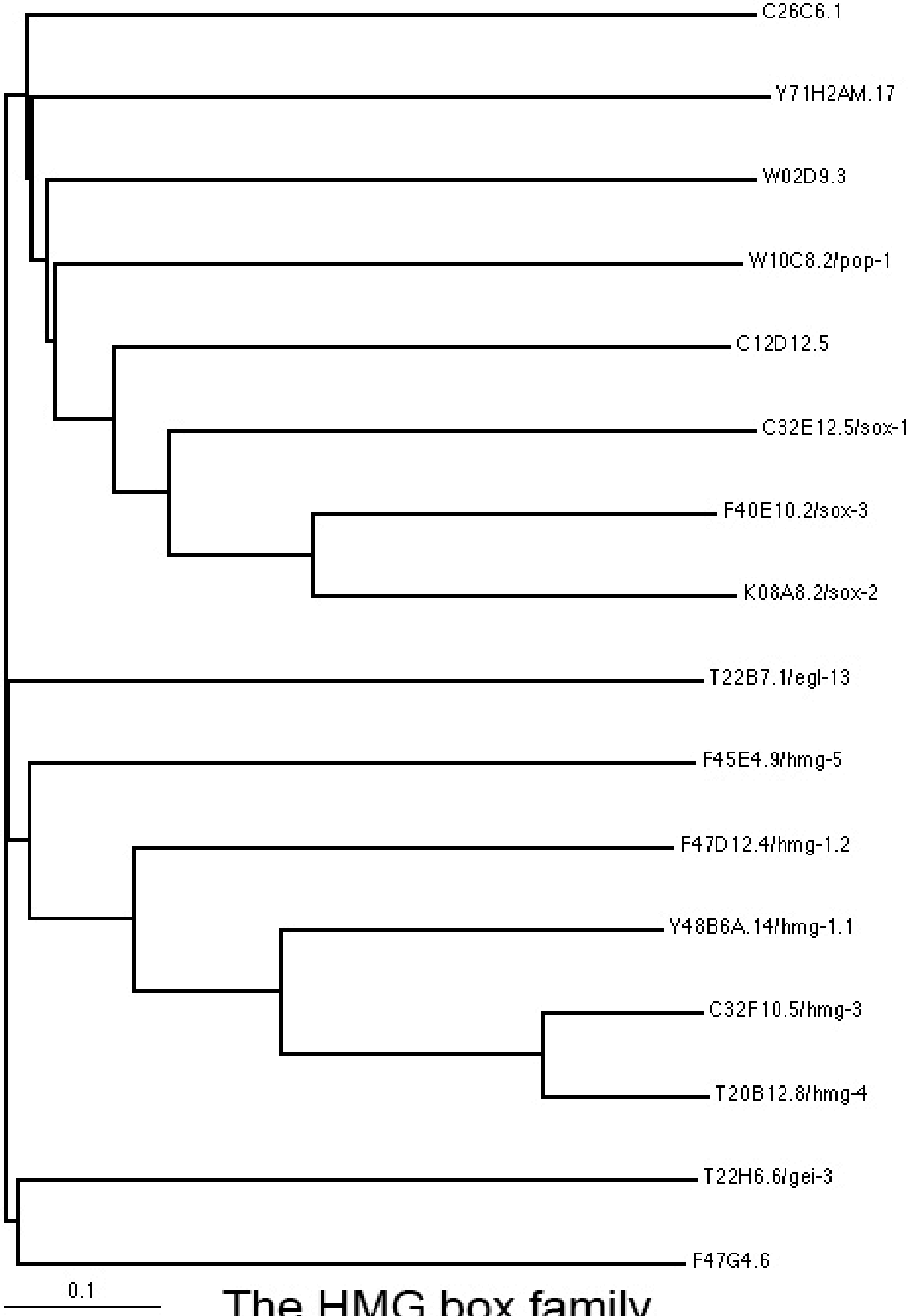

The HMG box family

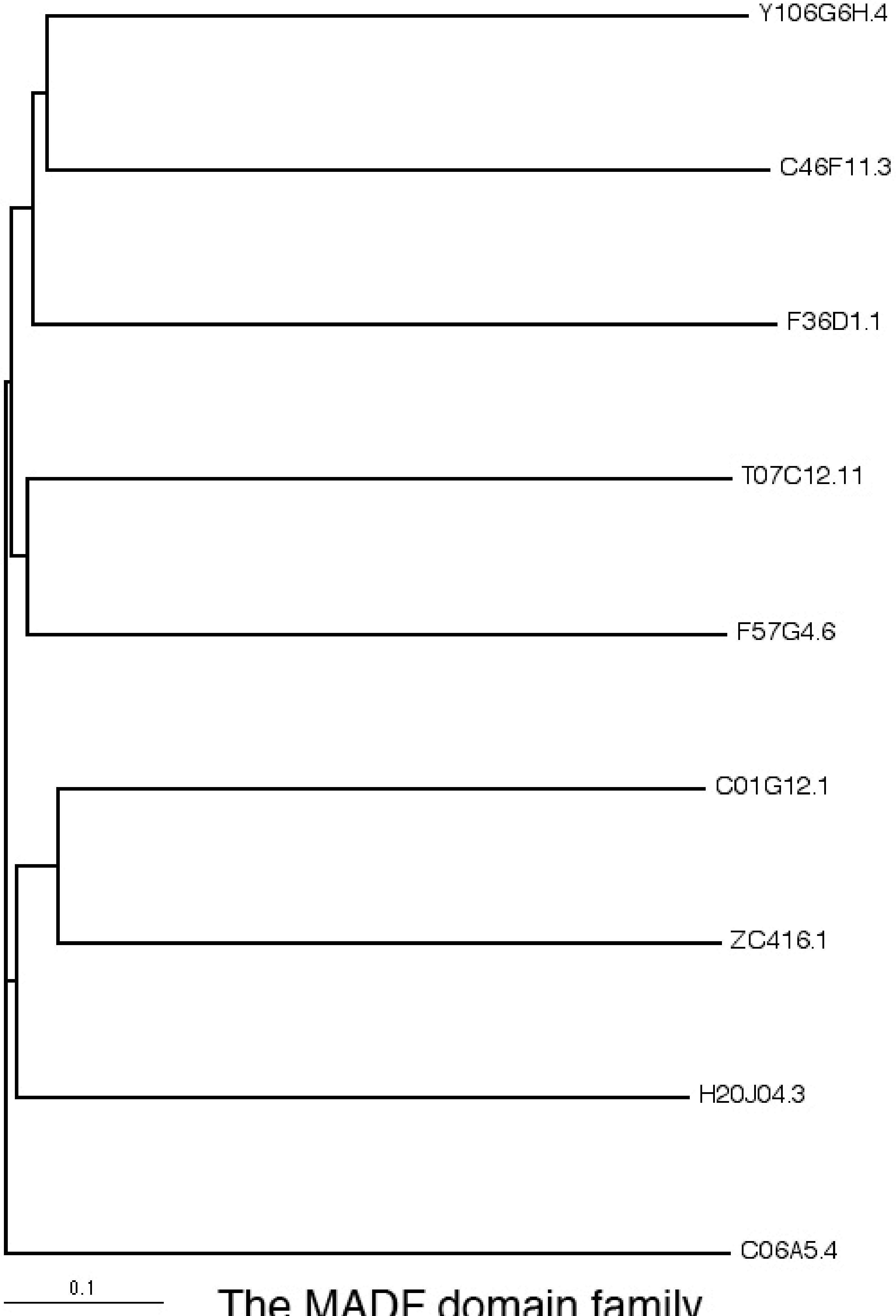

The MADF domain family

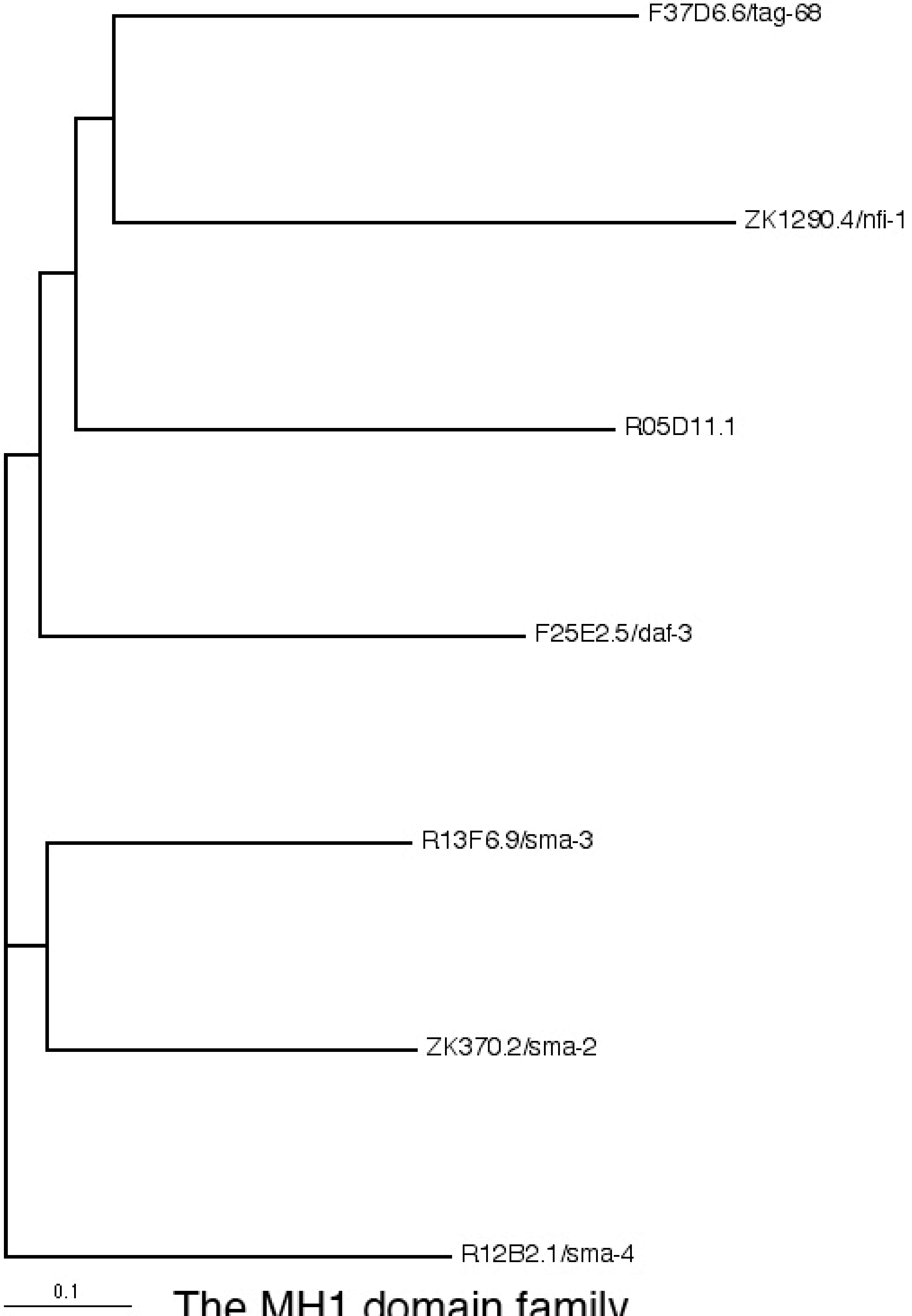

The MH1 domain family

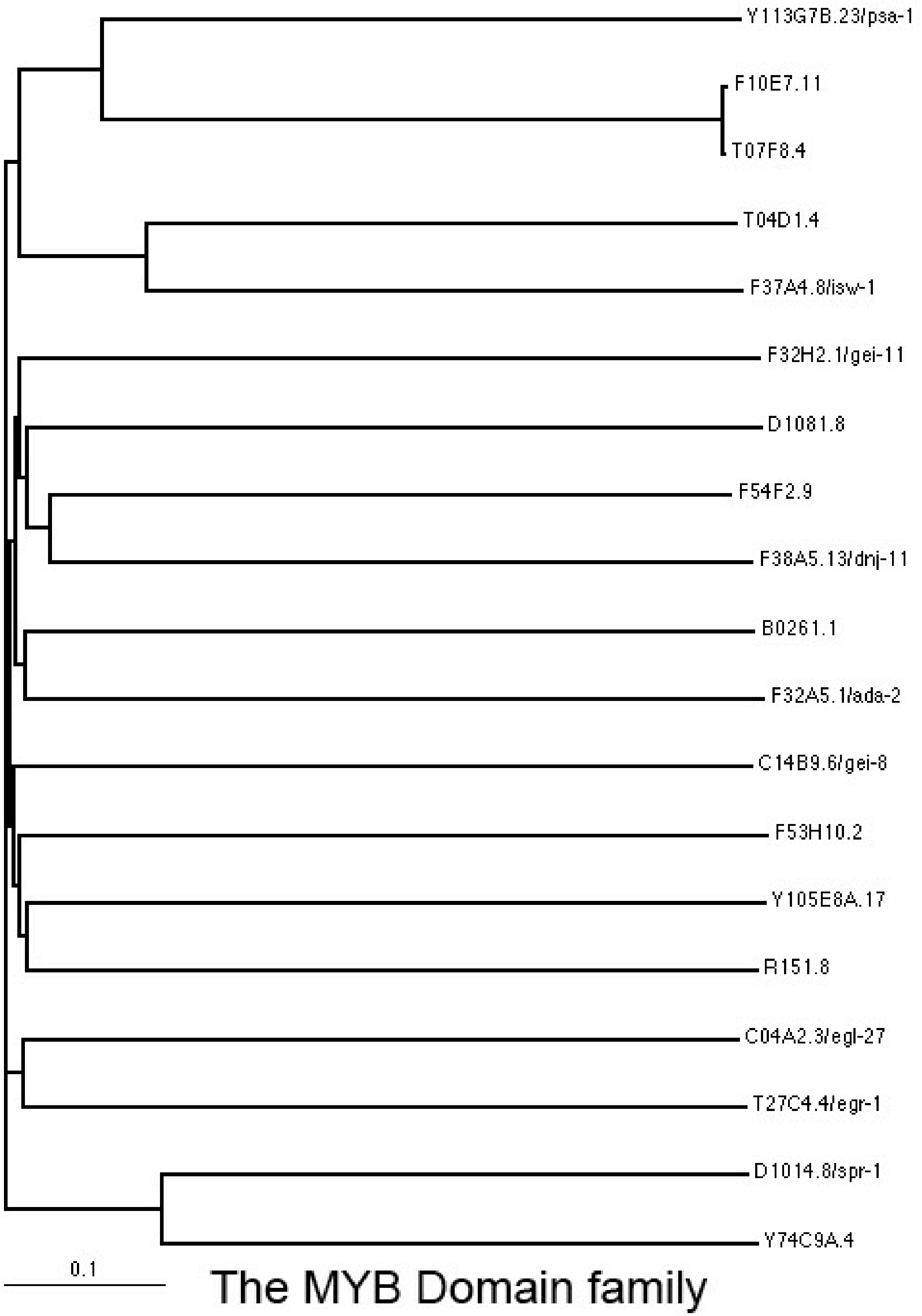

The MYB Domain family

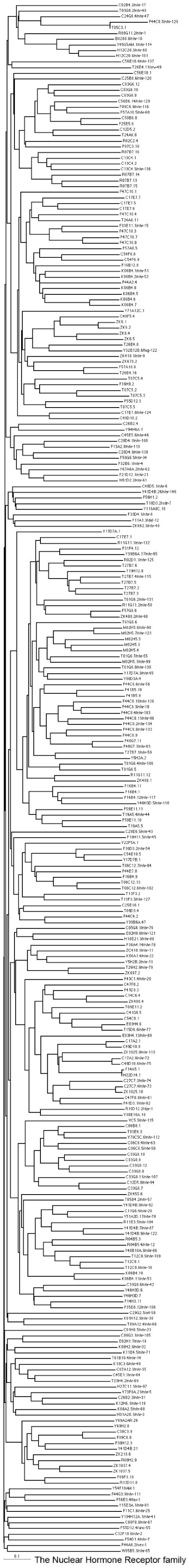

The Nuclear Hormone Receptor family

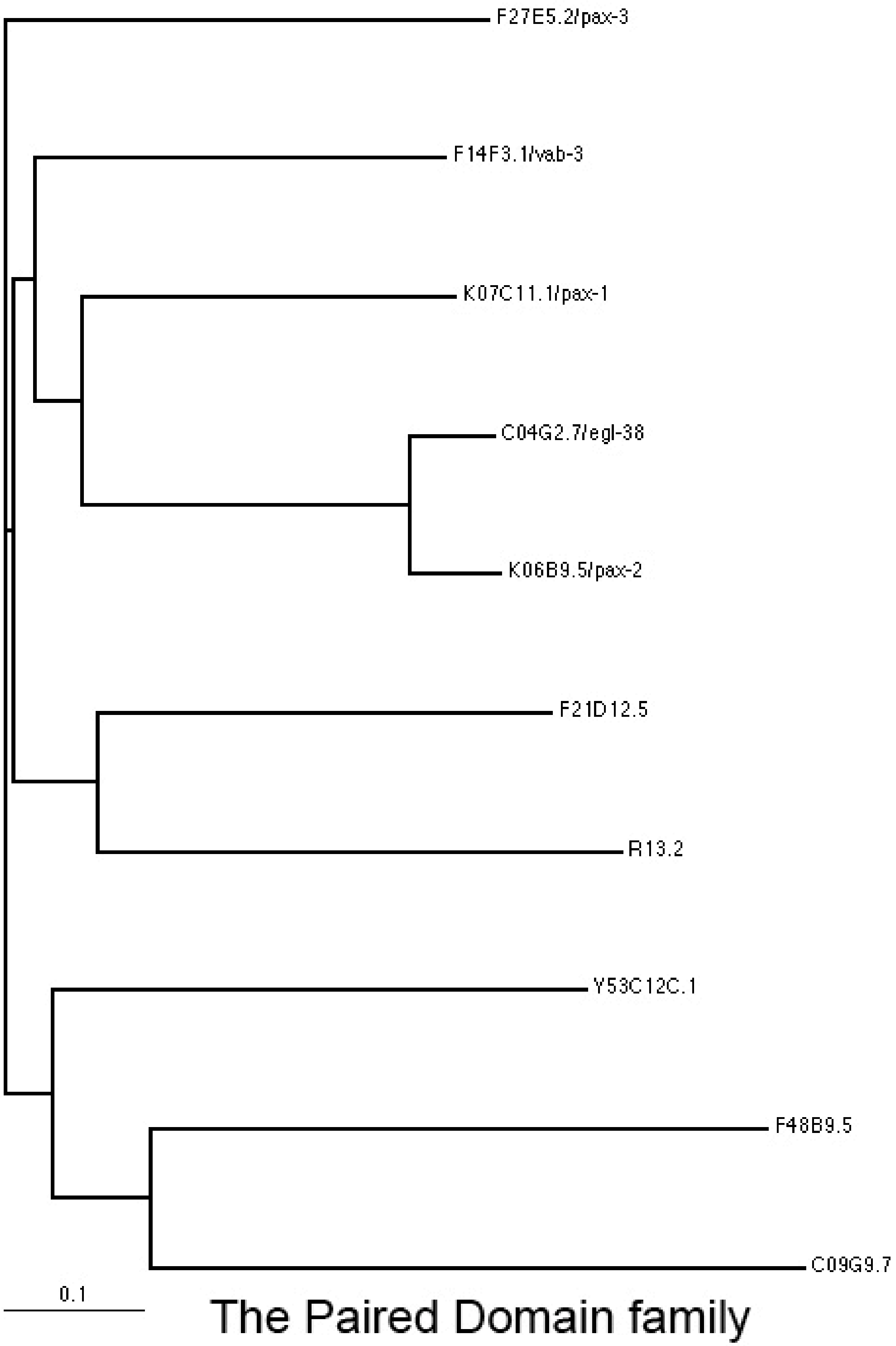

The Paired Domain family

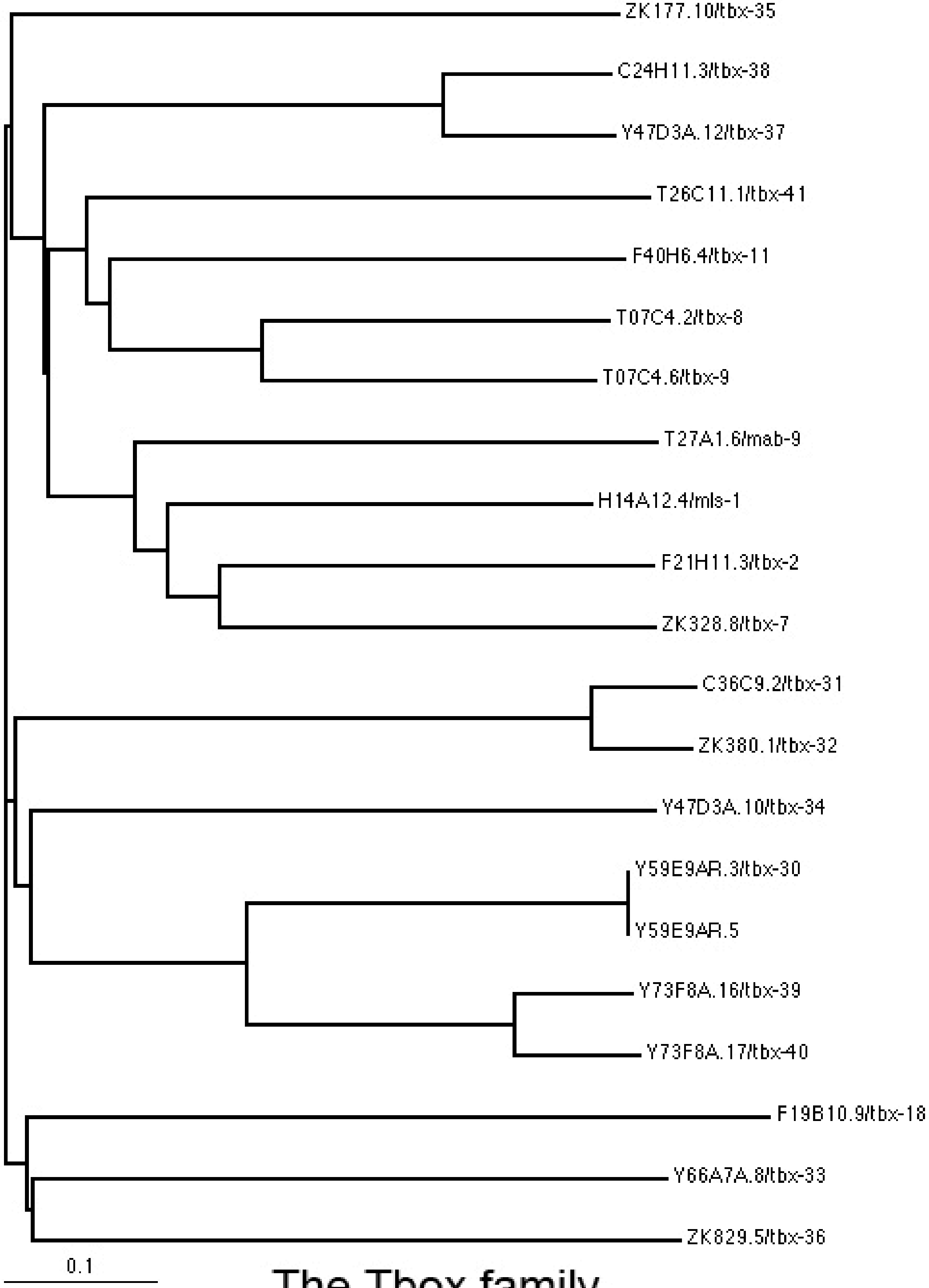

The Tbox family

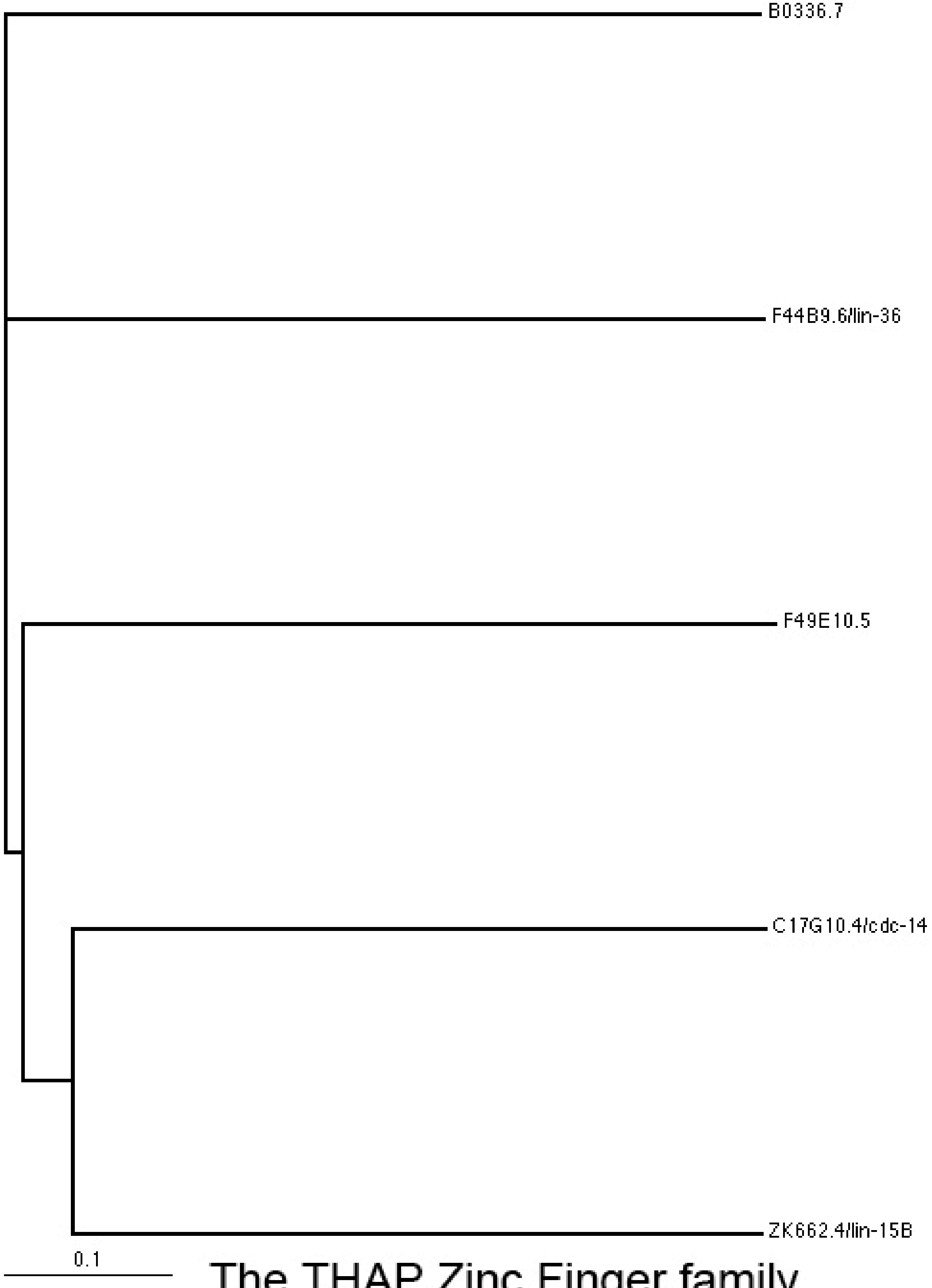

The THAP Zinc Finger family
